# Dual Roles of Cyanopeptides as Substrates and Inhibitors of Enzymes in Surface Waters

**DOI:** 10.1021/acs.est.6c07305

**Published:** 2026-09-29

**Authors:** Xuejian Wang, Elena Lippuner, Yaochun Yu, Elisabeth M. L. Janssen

**Affiliations:** † 28499 Swiss Federal Institute of Aquatic Science and Technology (EAWAG), 8600 Dübendorf, Switzerland; ‡ Department of Civil and Environmental Engineering, Stanford University, 473 Via Ortega, Stanford, California 94305, United States

**Keywords:** cyanobacteria, peptidase, hydrolysis, multicopper oxidase, oxidation

## Abstract

Harmful cyanobacterial blooms produce diverse bioactive metabolites, cyanopeptides, whose environmental persistence remains uncertain. First, we investigated enzymatic reactions with cyanopeptides as substrates in aquatic systems. The aquatic enzyme pool from the riverine biofilm showed peptidase and oxidase activities. Incubation of cyanopeptides with the natural aquatic enzyme pool revealed novel transformation products (TPs) of two microginins (nostoginin BN741 and microginin FR6) and TPs of anabaenopeptins that also formed when incubated with intact biofilm. Next, we probed underlying mechanisms with peptidases as functional analogs of the aquatic enzyme pool. Carboxypeptidase Y produced identical hydrolysis TPs of the two microginins. A multicopper oxidase rapidly transformed tyrosine-containing microcystins, anabaenopeptins, and microginins, in the presence of the mediator 2,2′-azinobis­(3-ethylbenzothiazoline-6-sulfonate) (ABTS). Tyrosine-free congeners remained stable. Lastly, we investigated cyanopeptides as enzyme inhibitors. Cyanopeptolins inhibited trypsin (IC_50_ = 53.75 nM), chymotrypsin (IC_50_ = 2.2 μM), and analogous activities in the aquatic enzyme pool. Anabaenopeptins and microginins showed only mild inhibition of carboxypeptidase Y. As different cyanopeptides are co-produced by cyanobacteria, biotransformation is expected to differ in mixtures compared to single-compound assessments due to the observed inhibition taking place at the same time. Similarly, their release during bloom events may influence the transformation of other organic compounds in surface waters.

## Graphical abstract

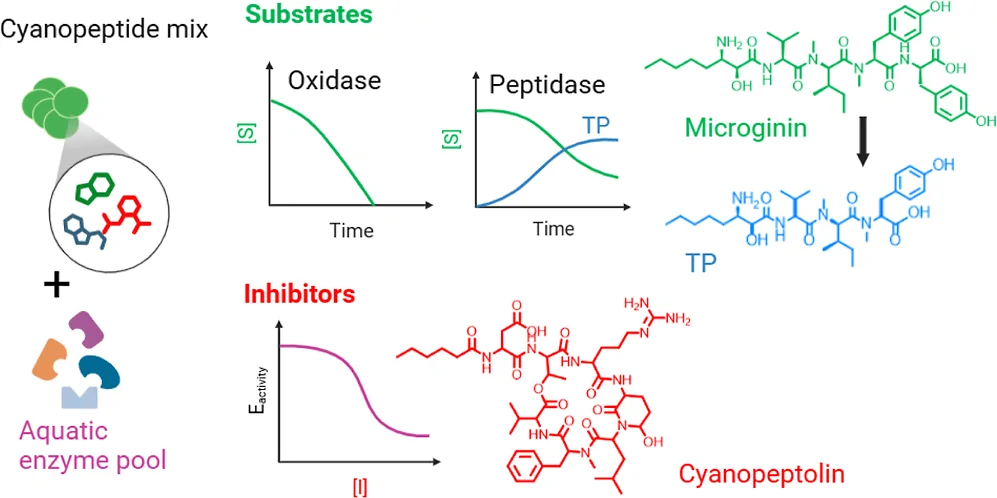

## Introduction

Harmful cyanobacterial blooms receive global attention as they are increasing in frequency, magnitude, and duration worldwide.^1^ One major concern is that they produce toxic compounds, such as microcystin-LR (MC-LR), anatoxin-a, saxitoxin, and cylindrospermopsin, which are recognized by the World Health Organization (WHO) as toxins.^2^ Besides these 4 WHO cyano-toxins, many more specialized metabolites are coproduced by bloom-forming cyanobacteria,^3–5^ and many new variants are reported each year.^6^ Based on structural similarities, the metabolites can be grouped into different classes, and many of them are peptide-based compounds, called cyanopeptides hereafter including microcystins, anabaenopeptins, cyanopeptolins, and microginins.^3,4^

Many cyanopeptides are reported to inhibit enzymes. For example, MC-LR binds to liver protein phosphatase (PP1 and PP2) causing dysregulation and forming of reactive oxygen species which can lead to liver failure.^7–10^ Anabaenopeptins are reported to inhibit phosphatases, human serine protease, and carboxypeptidases;^11–13^ cyanopeptolins can inhibit trypsin, chymotrypsin, and human serine protease;^14–16^ and microginins present an inhibition effect on leucine aminopeptidase and angiotensin-converting enzyme.^17,18^ While inhibitory effects of cyanopeptides on enzymes are known, these studies mainly focused on medicinal and human health-related enzymes. Studies of the effects on aquatic enzymes with cyanopeptides are only anecdotal. Inhibition of an aminopeptidase and a protein phosphatase by an apolar fraction of the cyanobacterial extracts was previously reported, and purified microcystins, aerucyclamide A, and cyanopeptolin A inhibit the aminopeptidase in the low mg/L range, while the phosphatase was less affected.^19^ Inhibition of aquatic enzymes can reduce nutrient and carbon turnover rates and may also impair metabolic functions of grazing organisms.

Considering the adverse effects of these cyanopeptides, it is important to know their fate in the environment to better assess their long-term effects on the ecosystem. Biotransformation is an important fate process for cyanopeptides in surface waters, especially in the absence of sunlight, when abiotic processes are not significant. Our previous study investigated biotransformation rates and transformation products (TPs) for 40 cyanopeptides, including microcystins, anabaenopeptins, cyanopeptolins, and microginins.^20^ The proposed transformation pathway analysis suggested that these reactions are carried out by aquatic enzymes. While studies of the transformation of cyanopeptides beyond microcystins remain limited, investigations into the underlying enzymatic transformation pathways are even scarcer. The enzymatic degradation of MC-LR has been best characterized: its cyclic structure is initially opened by microcystinase MlrA, after which the resulting linear peptide is further hydrolyzed by MlrB and MlrC.^21,22^ Peptide bond cleavage plays a central role in this process. Consistently, previous studies with the riverine biofilm and biofiltration during water treatment identified similar bond-cleavage products in anabaenopeptins, arising from the breakage of the C–N linkage between the cyclic core and the side chain.^20,23^ These studies suggest that hydrolytic reactions may be a common enzymatic pathway acting on cyanopeptides, given their peptide-based structures and the widespread occurrence of bacterial peptidases. Indeed, over 300 biochemically recognized peptidases have been reported in freshwater, and activities such as aminopeptidase (arginyl aminopeptidase, glycyl aminopeptidase, Leu-AP, and pyroglutamyl aminopeptidases) and trypsin-like enzymes were observed in 28 freshwater bodies.^24^ It remains to be evaluated which freshwater peptidases can effectively transform cyanopeptides.

In addition to peptide-bond cleavage, oxidation reactions can also contribute to cyanopeptide biotransformation. Our previous studies observed the oxidative transformation of tyrosine and tyrosine-like residues in microcystins and anabaenopeptins, involving the conversion of monophenols to diphenols.^20,23^ Another study indicated the possibility of epoxidation and hydroxylation of MC-LR by microbial communities.^25^ Research on enzymatic oxidation of cyanopeptides in aquatic environments is limited, and no study has examined the reactivity of cyanopeptides beyond microcystins with aquatic oxidases. Oxidases (oxidoreductases) are a diverse class of enzymes produced by aquatic microorganisms that catalyze the oxidation of a broad range of organic substrates including xenobiotic contaminants. These enzymes can contribute to environmentally relevant processes such as carbon cycling, energy metabolism, detoxification of reactive compounds, and biodegradation of natural organic matter and xenobiotic contaminants in surface water.^26,27^ Among them, bacterial laccases are widespread multicopper oxidases, with homologues frequently detected in freshwater microbial communities. Notably, a previous study demonstrated that a wastewater-relevant bacterial multicopper oxidase could transform a broad range of micropollutants, with significant removal observed for 38 compounds.^28^ Given their broad substrate promiscuity and prevalence in freshwater environments, bacterial laccases may therefore contribute to the oxidation and transformation of cyanopeptides in surface waters.

In this study, we explored the reactions of cyanopeptides as substrates for and inhibitors of aquatic-relevant enzymes. First, we focused on peptide bond hydrolysis and oxidation reactions and identified new TPs and confirmed previously known TPs by incubating cyanopeptides with an aquatic enzyme pool. We then used trypsin, chymotrypsin, and carboxypeptidase Y as functional analogs of some aquatic peptidases and examined their reactivity toward cyanopeptolins, anabaenopeptins, and microginins. We also tested a multicopper oxidase with microcystins, anabaenopeptins, cyanopeptolins, and microginins. In a second step, we tested whether cyanopeptides, including cyanopeptolins, anabaenopeptins, and microginins, inhibit aquatic enzymes, trypsin, chymotrypsin, or carboxypeptidase Y. Overall, we aimed to expand our understanding of different enzymatic reactions in the environmental fate process of cyanopeptides coproduced in harmful surface water blooms.

## Methods and Materials

### Enzymes

In this study, trypsin (T8003, Sigma-Aldrich), chymotrypsin (C3991, TCI-Chemical), and carboxypeptidase Y (C3888, Sigma-Aldrich) were used with the model substrates Z-l-Arg-7-amido-4-methylcoumarin hydrochloride (70375-22-3, Cayman Chemical), Ala-Ala-Phe-7-amido-4-methylcoumarin (A3401, Sigma-Aldrich), hippuryl-l-phenylalanine (H6875, Sigma-Aldrich), and hippuryl-l-arginine (H2508, Sigma-Aldrich), respectively (details in Table S1).

### Aquatic Enzymes

Periphyton biofilms were grown on glass slides in a water flow-channel supplied with a bypass from River Chriesbach in Switzerland as previously described.^20^ Briefly, the channel was operated at a constant flow rate of 0.1 m/s under a light/dark cycle of 12:12 h. The established biofilm material was removed from the glass after 4–6 weeks, suspended in stream water, and filtered (0.95 mm nylon net) to remove larger periphyton agglomerations and small organisms. Then the biofilm suspensions were sonicated (15 °C, 45 kHz, 80 W [power level 9, maximum output], 20 min, VWR USC300TH ultrasonic cleaner) to break the intact cells. The biofilm suspensions were then centrifuged (4000 *rcf*, 10 min, Eppendorf centrifuge 5910 Ri), and the supernatant was filtered through 0.22 μm filters. The freshly harvested aquatic enzyme pool was aliquoted and stored at −20 °C. The total protein content in the aquatic enzyme pool was measured with the Pierce BCA Protein Assy Kit (Thermo Fisher Scientific, details in Text S1).

### Oxidase

The multicopper oxidase (MCO1) previously identified from wastewater activated-sludge communities was used.^29^ The oxidase was mined directly from metatranscriptomic data, synthesized, expressed, and purified for functional testing. The oxidase is a laccase, which couples the reduction of molecular oxygen to the one-electron oxidation of a wide variety of aromatic substrates.^30^ The oxidase was incubated with cyanopeptide extracts with and without the presence of the redox mediator ABTS (2,2′-azino-bis­(3-ethylbenzothiazoline-6-sulfonic acid)).

### Cyanopeptide Extraction and Fractionation

Besides three microcystin standards (MC-LR, MC-LY, and MC-YR), cyanopeptide mixtures were extracted and fractionated from four cyanobacterial strain cultures including *Planktothrix rubescens* (K-0576) and *Microcystis aeruginosa* PCC7806 (wildtype), *M. aeruginosa* (*mcyD* knocked-out mutant), and *M. aeruginosa* (NPCD-01). Culture conditions and cyanopeptide extraction methods were carried out as described previously (Text S2).^20,31,32^

Methanolic extracts of cyanobacterial biomass were fractionated using an Ultimate 3000 UHPLC system (Thermo Scientific) coupled with a FoxyJr Fraction collector. A kinetex liquid chromatography (LC) column (C18, 5 μm particle size, 100 Å pore size, 150 × 4.6 mm, Phenomenex) fitted with an in-line filter (BGB) was used and maintained at 40 °C. The mobile phases consisted of nanopure water (A) and methanol (B) both acidified with formic acid (0.1%). Binary gradient elution was carried out at a flow of 1.2 mL/min, increasing eluent B steadily from 20%. Fractions were collected for each minute separately from 2nd min to 25th min. The methanol content of the collected fractions was first dried under a gentle stream of N_2_ in a 40 °C water bath (Turbovap LV, Biotage, Sweden) and then diluted with nanopure water until content reached below 2%. Solid-phase extraction was conducted to remove formic acid and further purify the fractions, the trapped sample was washed with nanopure water and 20:80% v/v methanol: water, and eluates were collected in 85:15% v/v methanol/water and stored at −20 °C for further analysis. The methanol in the fractions was evaporated with N_2_ in a 40 °C water bath before enzyme interaction experiments.

### Cyanopeptide Analysis

Cyanopeptide extracts and fractions were analyzed by high-performance liquid chromatography (HPLC, Dionex Ultimate 3000 RS pump, Thermo Fisher Scientific) coupled with high-resolution tandem mass spectrometry (HRMS/MS, Fusion Lumos Thermo Fisher Scientific).^20^ The injection volume was 100 μL, and separation was carried out with a Kinetex C18 column (2.6 μm, 2.1 × 100 mm, Phenomenex, precolumn VanGuard Cartridge, Waters). And two mobile phases included nanopure water (A) and methanol (B) both acidified with formic acid (0.1%), and the binary gradient elution was carried out at a flow rate of 0.255 mL/min, increasing eluent B steadily from 20% to 70% between 0 and 30 min, from 70% to 100% between 30 and 31.4 min, and keeping at 100% between 31.4 and 38.5 min, then decreasing from 100% to 20% between 38.5 and 40 min, and keeping at 20% until 45 min. HRMS/MS used electrospray ionization (ESI) with 320 °C capillary temperature, 3.5 kV electrospray voltage, 275 °C vaporizer temperature, positive ionization mode, full scan from 120 to 1200 *m*/*z* with a nominal resolving power of 120,000 at *m*/*z* 200, 25% automated gain control (AGC) target, 50 ms maximal injection time. Data-dependent high-resolution product ion spectra were obtained by normalized collision energies for HCD of 15%, 30%, and 45% at a resolving power of 15,000 at 200 *m*/*z* isolation window, triggering data-dependent MS/MS acquisition using the public database CyanoMetDB.^3,33^

Data analysis and peak area extraction were performed with Skyline 25.1 (MacCoss Lab Software).^20^ The identification was based on exact mass (<5 ppm mass error), accurate isotopic pattern of the precursor ion (Skyline idotP > 0.95 considering top three isotopes), and match of retention time and MS^2^ spectra of reference materials. Suspect screening analysis was also conducted for cyanopeptides, for which no reference material was available. Therefore, full-scan (MS^1^) spectra were screened for exact masses (<5 ppm mass error) and accurate isotopic patterns of the precursor ion (Skyline idotP > 0.95) for cyanopeptides reported in the cyanobacterial suspect list CyanoMetDB.^3,33^ Confidence in identification followed the Schymanski et al. scheme:^34^ metabolites were classified as tentative candidates (Level 3) based on exact mass (<5 ppm mass error), accurate isotopic pattern (Skyline idotP value >0.95), and evidence from fragmentation data; as probable structures (Level 2b) based on additional, comprehensive MS^2^ fragmentation information that helped to confirm the connectivity of molecular substructures; or as confirmed structures (Level 1) when these parameters were in agreement with available reference standards or bioreagents (Table S2). Documentation of the annotation of cyanopeptides has been previously published,^31,35^ and cyanopeptides and confidence levels are listed in Table S3. External 10-point calibrations were included for 18 cyanopeptides for which commercial reference materials were available (Table S2) at the beginning and at the end of each MS sequence. A serial dilution of extracts was used to verify the linear instrumental response of other cyanopeptides, and semiquantification with the structurally most similar reference material was performed (Table 1 footnotes).

**Table 1:** Cyanopeptide Concentrations (μM) in Fractions from Different Cyanobacterial Strains^a^

strain	Concentration (μM)	
M. aeruginosa (*mcy*D knocked-out mutant)	M-1	
Cyanopeptolin A	115.54	
Cyanopeptolin B	26.64^b^	
Cyanopeptolin C	18.79^b^	
Cyanopeptolin D	61.03	
Aerucyclamide A	10.52	
*sum cyanopeptolin*	222.00	
**P. rubescens**	**P-1**	**P-2**
Anabaenopeptin A	0.64	126.40
Anabaenopeptin B	41.46	1.39
Oscillamide Y	1.43	1.48
Anabaenopeptin F	0.93^b^	0.90^b^
Anabaenopeptin SA13	0.63^b^	0.67^b^
Oscillapeptin J	1.45^b^	1.20^b^
[d-Asp,^3^(*E*)-Dhb^7^MC-RR	2.40	2.43
*sum anabaenopeptin*	45.09	130.84
M. aeruginosa **(NPDC-01)**	**M-2**	
Nostoginin BN741	1314.94^b^	
Microginin FR6	31.77^b^	
Cyanopeptolin 959	18.22^b^	
*sum microginin*	1346.71	

a P1 and P2 from *P. rubescens* (K-0576), M-1 from *M. aeruginosa* (PCC7806 *mcy*D knocked-out mutant), and M-2 from *M. aeruginosa* (NPCD-01).

b Note: The concentration of anabaenopeptin F is expressed as oscillamide Y equivalent concentration; that of anabaenopeptin SA13 as anabaenopeptin A equivalent concentration; those of cyanopeptolins B and C and cyanopeptolin 959 as cyanopeptolin A equivalent concentration; and those of nostoginin BN741 and microginin FR6 as MC-LR equivalent concentration.

### Incubation of Enzymes with Cyanopeptides

The reactivity of cyanopeptides with the aquatic enzyme pool, trypsin, chymotrypsin, carboxypeptidase Y, and oxidase was assessed by monitoring cyanopeptide concentrations by HPLC-HRMS/MS during incubation experiments. Combinations of enzymes and cyanopeptides are detailed in Table S4. Control setups included cyanopeptide exposure in nanopure water and autoclaved biofilm suspensions. For the aquatic enzyme pool and the peptidases, tests were carried out in triplicate glass vials covered with sterilized cotton plugs and placed on a vertical shaker with a UV-light filter (226, LEE filters, Hampshire, UK) to exclude direct photochemical transformation. Each vial was subsampled (300 μL) over the course of 4 days, and samples were immediately stored in glass vials at −20 °C until further cyanopeptide analysis. For the oxidase, due to limited amount of enzyme materials, 96-well plates were used. For each well, oxidase, ABTS, and cyanopeptides (extraction/fractions/standards) were mixed in ammonium acetate buffer (pH ∼ 5.5) for the active group at a final volume of 250 μL. Enzyme control (no ABTS added) and ABTS control (no oxidase added) were also conducted. A breathing-membrane was used to cover the wells to slow down the water evaporation, and the 96-well plates were incubated at 30 °C on a shaker for 2 days. For sub-sampling, 20 μL samples were mixed first with 50 μL of methanol to stop the enzyme reaction and then diluted with 130 μL of buffer. The samples were stored at 4 °C shortly before instrument analysis.

### Enzyme Inhibitory Effect

The activity of commercial peptidases (trypsin, chymotrypsin, and carboxypeptidase Y) and the aquatic enzyme pool was expressed as a product formation rate using model substrates (Text S3). A wide range of trypsin and chymotrypsin concentrations were considered for inhibition tests guided by previous studies (Table S5). This range of inhibitory effects may be due to different activities of cyanopeptide variants, different origins of enzyme materials, and different choices of model substrates. Based on Michaelis–Menton kinetics and instrument response for rate calculation, the trypsin (50 nM) and chymotrypsin (70 nM) concentrations were selected. Inhibitory effects were evaluated after pre-incubation of cyanopeptides with the enzymes (30–40 min). A shoulder-log model was fitted from the ratio of the reaction rate in the presence of cyanopeptides *V*_C_ (μM/s) relative to the measured reaction rate in control, i.e., without incubating with cyanopeptides *V*_0_ (μM/s), with cyanopeptide concentration *C* (μM); the shoulder or lag concentration where minimal inhibition of the enzyme was detected *S* (μM); and *k* (μM^–1^) the inhibition constant for the log linear portion of the inhibition curve:

$$\frac{V_{c}}{V_{0}}=e^{-kC}\left(\frac{e^{kS}}{1+\left(e^{kS}-1\right)e^{-kC}}\right)$$

Nonlinear least-squares fitting was performed using the *nlsLM* function in R (2024. 12.0 + 467). When possible, the IC_50_ values were calculated for *V*_C_/*V*_0_ equal to 0.5.

### Cyanopeptide Degradation and Transformation Products

Decay kinetics of cyanopeptide-identified TPs were followed by calculating the relative abundance for all subsampling time points as *A*_t_/*A*_0_, with measured areas (*A*) at time *t* compared to the initial area at time 0. The transformation products were investigated using a previously published approach when similar cyanopeptides were incubated with biofilm suspensions.^20^ Previously identified TPs from the biofilm incubation and *in silico* predictions from the SMILES of parent compounds using EnviPath and BioTransformer 3.0 were considered. To identify TPs in experimental samples, suspect screening of the collated list of possible TPs was applied. MS^2^ spectra annotation and assignment of the confidence level were conducted based on the above-mentioned criteria. Peak areas of selected ion chromatograms of TPs were also extracted for kinetic analysis. Any TPs present in the autoclaved controls were excluded from further analysis.

## Results and Discussion

### Cyanopeptides as Enzyme Substrates

In this study, the transformation of cyanopeptides by enzymes was examined. First, cyanopeptides were incubated with an aquatic enzyme pool extracted from biofilm suspensions previously grown *in situ* in river water. TPs resulting from peptide bond (-like) hydrolysis and tyrosine oxidation were identified. Most TPs and formation pathways have previously been comprehensively characterized.^20^ In this study, we incubated cyanopeptides with selected enzymes directly to focus on the specific roles of peptidases and oxidases in these transformations.

### Peptide-Bond Hydrolysis

The enzyme pool from surface water biofilm suspensions contained a protein equivalent of 174 μg_BSA_/mL. First, hydrolysis of model substrates and the production of corresponding products confirmed the presence of functional analogs of trypsin, chymotrypsin, and carboxypeptidase Y activity in the aquatic enzyme pool. Michaelis–Menten kinetics for trypsin, chymotrypsin, and carboxypeptidase Y and quasi-Michaelis–Menten kinetics for the aquatic enzyme pool with the chymotrypsin substrate are shown in Figure S1. Next, 4 days of exposure to the aquatic enzyme pool demonstrated the decay of cyanopeptides for cyanopeptolins A–D, anabaenopeptins A–B, and oscillamide Y (Figure 1a). Abiotic controls (autoclaved) showed no or significantly less decay (Figure 1b). Novel hydrolysis TPs of two microginins (nostoginin BN741 and microginin FR6; Figure 2a,b) were identified along with previously reported peptide bond-like hydrolysis products of anabaenopeptins (Figure S2).^20^ The TP of each microginin resulted from hydrolysis of the peptide bond of the C-terminal tyrosine that was cleaved off the parent structures, as highlighted in red in Figure 2. Annotation of the MS^2^ spectra suggests that the confidence level of the TP of nostoginin BN741 is level 2b (Figure 2c) and level 3 for the microginin FR6 product with less clear proof by the available MS^2^ fragments. Although the relative decay of nostoginin BN741 and microginin FR6 was minor for the 4 day exposure (*t*-test, ρ > 0.05), the increase of their corresponding TPs was significant (*t*-test, ρ < 0.05), due to a relatively high starting concentration of the parent compounds. No hydrolysis product of cyanopeptolins was observed in the aquatic enzyme pool.

**Figure 1:**
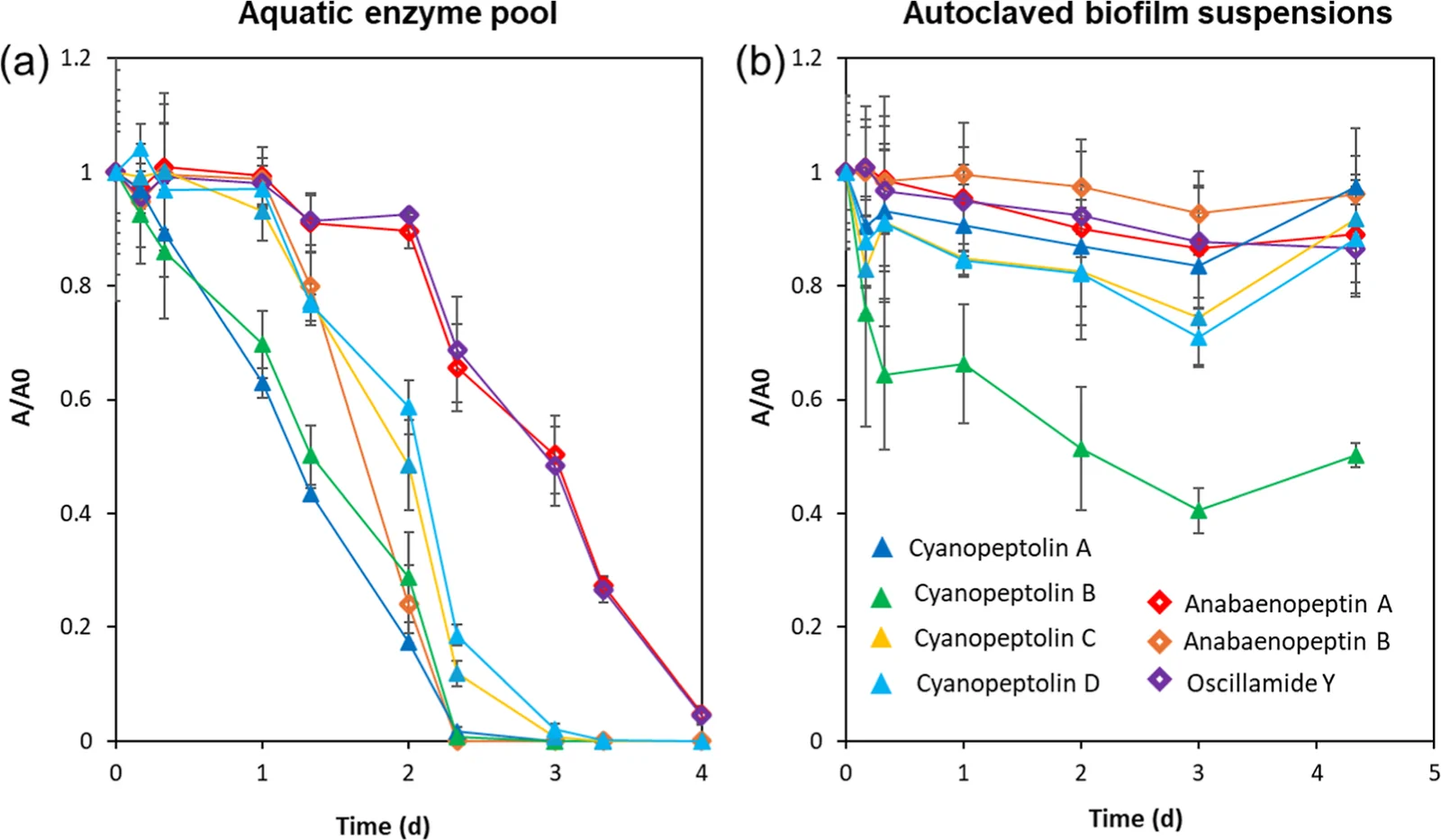
(a) Decay kinetics of cyanopeptides in the aquatic enzyme pool calculated from the peak area at each sampling point normalized by the initial peak area (*A*/*A*_0_) and (b) autoclaved biofilm suspension controls.

**Figure 2:**
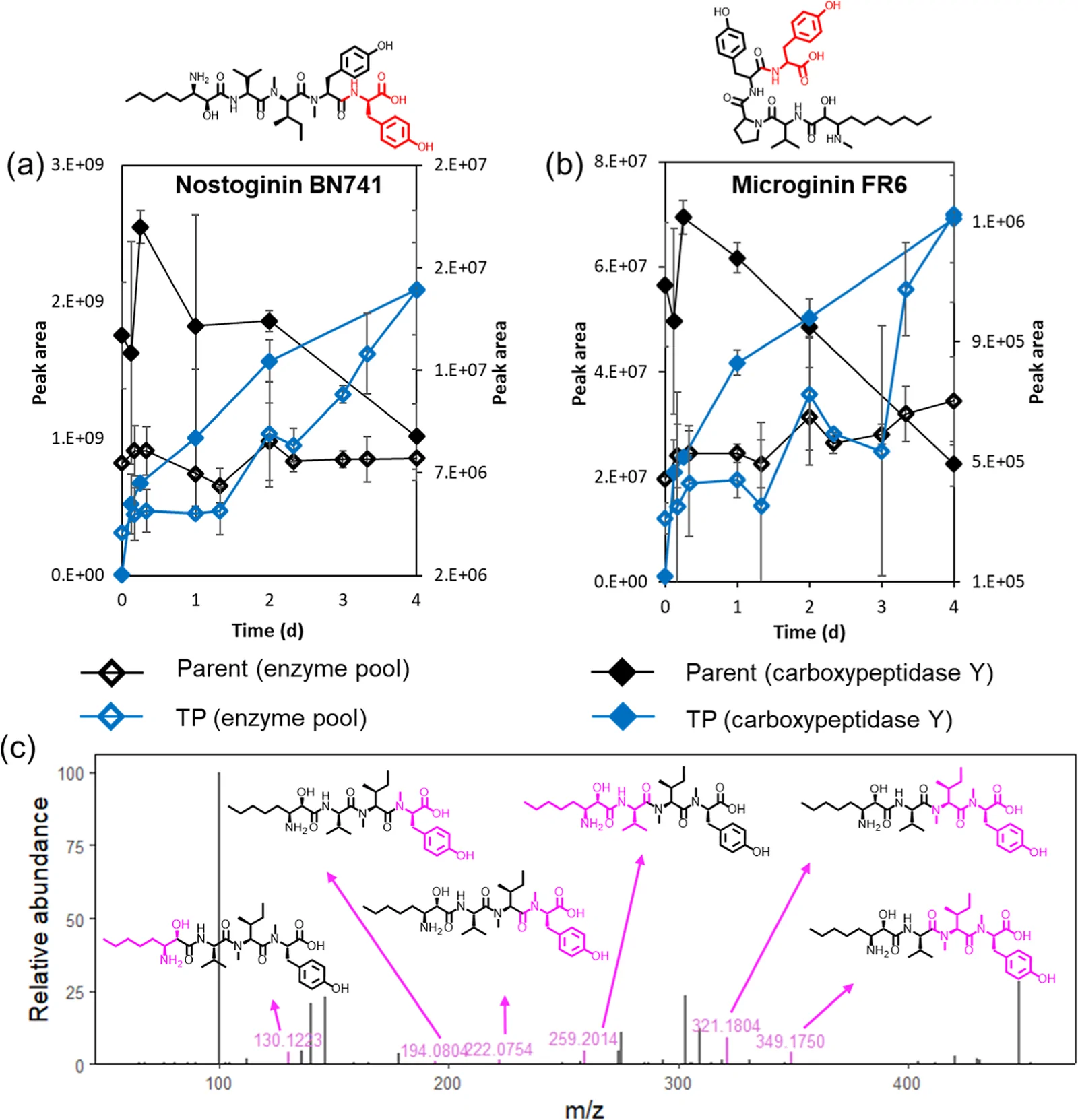
Concentration change (as peak area) of (a) nostoginin BN741 and (b) microginin FR6 during exposure to the aquatic enzyme pool (black open diamonds) and carboxypeptidase Y (black diamonds), and formation of the same hydrolysis product in the aquatic enzyme pool (blue open diamonds) and incubated with carboxypeptidase Y (blue diamonds). The peak areas of TPs in the aquatic enzyme pool are shown on the secondary *y*-axes. (c) Annotation of MS^2^ spectrum of the transformation product of nostoginin BN741.

As such an aquatic enzyme pool contains a complex mixture of enzymes, one would need to identify, isolate, enrich, and test the peptidases that are responsible for cyanopeptide degradation. Instead, we focused on selected peptidase functions based on the observed TPs. We used commercially available peptidases as functional analogs of part of the aquatic peptidase pool to further explore the reaction mechanisms. We selected trypsin, chymotrypsin, and carboxypeptidase Y because we identified that several cyanopeptolins, anabaenopeptins, and microginins can potentially be suitable substrates. Previously, microcystins did not show hydrolysis TPs that are expected to be linked to these peptidases and were hence not further assessed herein.^20^ Incubation with carboxypeptidase Y resulted in the same hydrolysis TPs of the two microginins as observed with the aquatic enzyme pool (nostoginin BN741 and microginin FR6, Figure 2A,B). Again, the decay of the parent compound nostoginin BN741 after exposure to carboxypeptidase Y was not significant (*t*-test, ρ > 0.05) during a 4 day exposure, yet the increase of corresponding TP was significant (*t*-test, ρ < 0.05). The decay of microginin FR6 and increase of corresponding TP were both significant (*t*-test, ρ < 0.05, Figure 2A,B). The observed reactions with two microginins agreed with the preference of carboxypeptidase Y to cleave tyrosine at the C-terminus. Based on its cleavage preference, the TPs formed are themselves potential substrates for carboxypeptidase Y. However, no further hydrolysis of these TPs was observed, neither with carboxypeptidase Y nor with the aquatic enzyme pool. Methylated nitrogen in the peptide bonds can increase the stability against trypsin and chymotrypsin digestion.^36^ A similar effect may apply for the stability against carboxypeptidase Y digestion, which would explain the persistence of the TP of nostoginin BN741 but not for microginin FR6. Overall, the result demonstrates that aquatic enzymes can successfully degrade these and structurally similar microginins by the presence of carboxypeptidase Y-like activity.

Except for the hydrolysis of microginins by carboxypeptidase Y, no reaction was observed between the other cyanopeptides (cyanopeptolins A–D, anabaenopeptins A–B, and oscillamide Y) and any of the three peptidases. As trypsin preferentially hydrolyzes peptide bonds with a basic amino acid (e.g., lysine and arginine) on the carboxyl side, and chymotrypsin preferentially hydrolyzes peptide bonds with an aromatic amino acid (e.g., phenylalanine, tyrosine, and tryptophan) at this position, 4 cyanopeptolins and 3 anabaenopeptins could have been potential substrates (Figure S3). Several factors may explain the lack of trypsin and chymotrypsin activity toward these cyanopeptides. First, the cyclic structure may hinder the interaction of cyanopeptides with the active site of the enzymes. A previous study compared the stability of a cyclic peptide and its linear form when bound to a signaling protein, and substantially enhanced proteolytic stability of the cyclic form compared to the linearized form was observed.^37^ In fact, cyclic peptides show higher potential in drug delivery because of their enhanced stability compared to linear peptides.^38,39^ Second, the peptidases may not hydrolyze the peptide bonds of targeted amino acid residues if the residues are modified or the peptide bonds are modified (e.g., *N*-methylation),^36^ which is the case for the C-terminal amino acid of the anabaenopeptins. In summary, although the aquatic enzyme pool contains functional analogs of trypsin and chymotrypsin and can cleave their model substrates, the observed peptide bond-like hydrolysis of anabaenopeptins resulted from other peptidases with the same hydrolysis preference underlining the diversity of peptidase activities in the aquatic environment.

### Oxidation Reaction

In addition to TPs from peptide bond hydrolysis, previously reported tyrosine oxidation products^20^ were also observed during the incubation of cyanopeptides with the aquatic enzyme pool. To examine the reactivity of cyanopeptides with aquatic oxidases, we synthesized and heterologously expressed a multicopper oxidase based on activated sludge metatranscriptomic data.^29^ A previous study verified the prevalence of the oxidase homologues in freshwater systems such as lakes and sediments, indicating the presence of functional analogs in the aquatic environment.^28^

Transformation of 14 cyanopeptides in the presence of the oxidase was monitored covering several structural classes, namely, microcystins, anabaenopeptins, microginins, cyanopeptolins, and cyclamides (Figure 3). Seven cyanopeptides showed significant decay within 1 to 2 days (i.e., anabaenopeptins A–B, oscillamide Y, nostoginin BN741, microginin FR6, MC-LY, and MC-YR), whereas the other seven cyanopeptides were rather stable (i.e., MC-LR, [d-Asp^3^MC-LR, cyanopeptolin A, cyanopeptolin D, cyanopeptolin 959, aerucyclamide Y, and planktocyclin). All seven labile cyanopeptides contain tyrosine (-like) residues in their structures (Figure S4), while the other seven cyanopeptides that remained stable lack such residues. The tyrosine-dependent decay can be clearly illustrated by comparing results for MC-LR to MC-LY and MC-YR, for which reference materials were used in addition to extracts from cyanobacterial biomass. These microcystins only differ in one amino acid residue and MC-LR was stable, whereas the two tyrosine-containing MC-LY and MC-YR were labile (Figure 3, middle row). These results demonstrate, for the first time, that an oxidase can use tyrosine-containing cyanopeptides as substrates.

**Figure 3:**
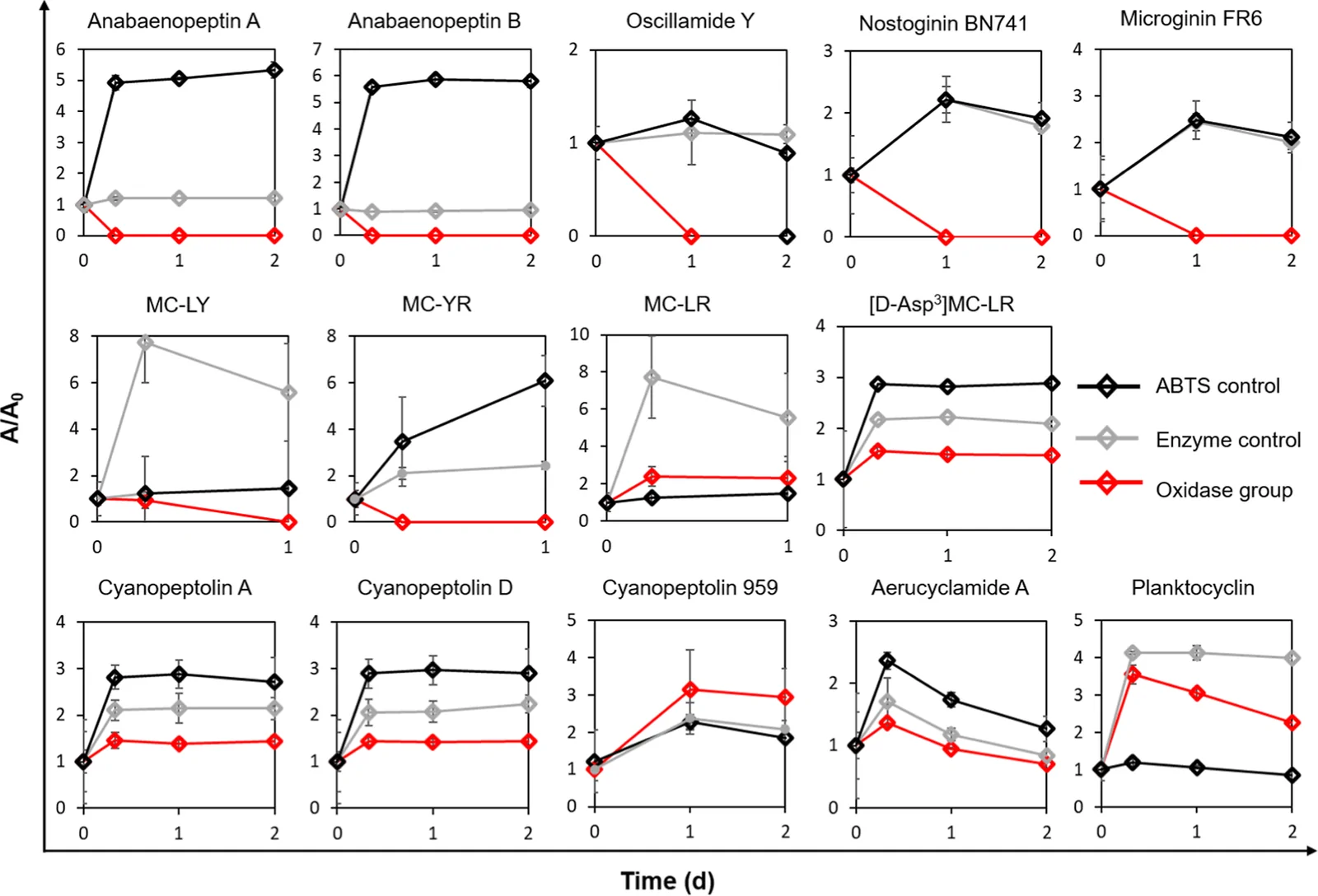
Decay kinetics during exposure to the active laccase oxidase, i.e., the active group (oxidase group, red), to MCO1 without ABTS (enzyme control, gray), and to ABTS group without the oxidase (ABTS control, black) for anabaenopeptin A, anabaenopeptin B, oscillamide Y, nostoginin BN741, microginin FR6, MC-LR, [d-Asp^3^MC-LR, MC-LY, MC-YR, cyanopeptolin A, cyanopeptolin D, cyanopeptolin 959, aerucyclamide A, and planktocyclin. *Y*-axis is expressed as relative concentrations (*A/A*_*0*_) calculated as peak area at each sampling time (*A*) relative to the initial peak area (*A*_*0*_).

No cyanopeptide showed significant decay in the controls, when only the oxidase (enzyme control) or only ABTS (ABTS control), indicating that ABTS, as the redox mediator, is required for effective oxidase-mediated cyanopeptide biotransformation. The stability of cyanopeptides when exposed only to the oxidase highlights the essential role of ABTS as a mediator in driving the oxidation. While ABTS is unlikely to be present in aquatic environments, other natural aquatic compounds can act as redox mediators such as phenolic and aromatic compounds in dissolved organic matter.^40–42^ Note that these control groups did show an increase in the relative concentration of cyanopeptides, which we attribute to the experimental circumstances (Figure 3). Miniaturized setups were used due to the limited amount of oxidase available, which led to volume loss due to evaporation despite best efforts by employing breathing membranes to cover reactions vessels. Nevertheless, the stark difference between fast degradation in the active group compared to the stable or increasing concentration in the controls supports our conclusions.

Laccases are known to oxidize phenolic compounds even without mediators, for example, phenolic lignin subunits.^30,43^ The fact that, in our system, no cyanopeptide degraded when incubated only with the oxidase could be due to the sterically constrained cyclic structure of cyanopeptides, which may limit direct enzyme–substrate interactions. Beyond tyrosine, previous studies have shown differences in the reactivity of the ABTS radical with amino acids, suggesting that cysteine reacted the fastest, followed by tryptophan and tyrosine, then histidine, while cystine reacted the slowest.^44^ In our study, only tyrosine was present in the 14 cyanopeptides. Since tyrosine oxidation was observed during the incubation of anabaenopeptins with the aquatic enzyme pool, this suggests that oxidation of tyrosine (-like) residues in cyanopeptides may play an important role in their environmental fate in surface waters. In this study, we were not able to identify any oxidation products with the oxidase; thus, we cannot conclude whether the cyclic structure of cyanopeptides remained intact. Oxidation of tyrosine moieties in cyanopeptides will change their polarity and affect their water chemistry; however, it is not immediately clear how this may affect their bioactivity, e.g., the toxicity of microcystins, which warrants further study.

### Cyanopeptides as Enzyme Inhibitors

Cyanopeptides are known to inhibit enzymes, and studies have focused on enzymes of human or animal origin. In Figure 4, we illustrate reported half-maximal inhibitory concentrations (IC_50_) of various enzymes by variants of nine cyanopeptide classes (also Table S5). However, cyanopeptides can also inhibit aquatic enzymes, for example, bacterial leucine aminopeptidases can be inhibited by microcystins, aerucyclamide A, and cyanopeptolin A.^19^ During cyanobacterial bloom events, the potential inhibitory effects of cyanopeptides on aquatic enzymes will, on the one hand, slow the degradation of organic substrates and thus influence the ecosystem functioning. At the same time, inhibition of enzymes may also slow the degradation of some toxins and micropollutants.

**Figure 4:**
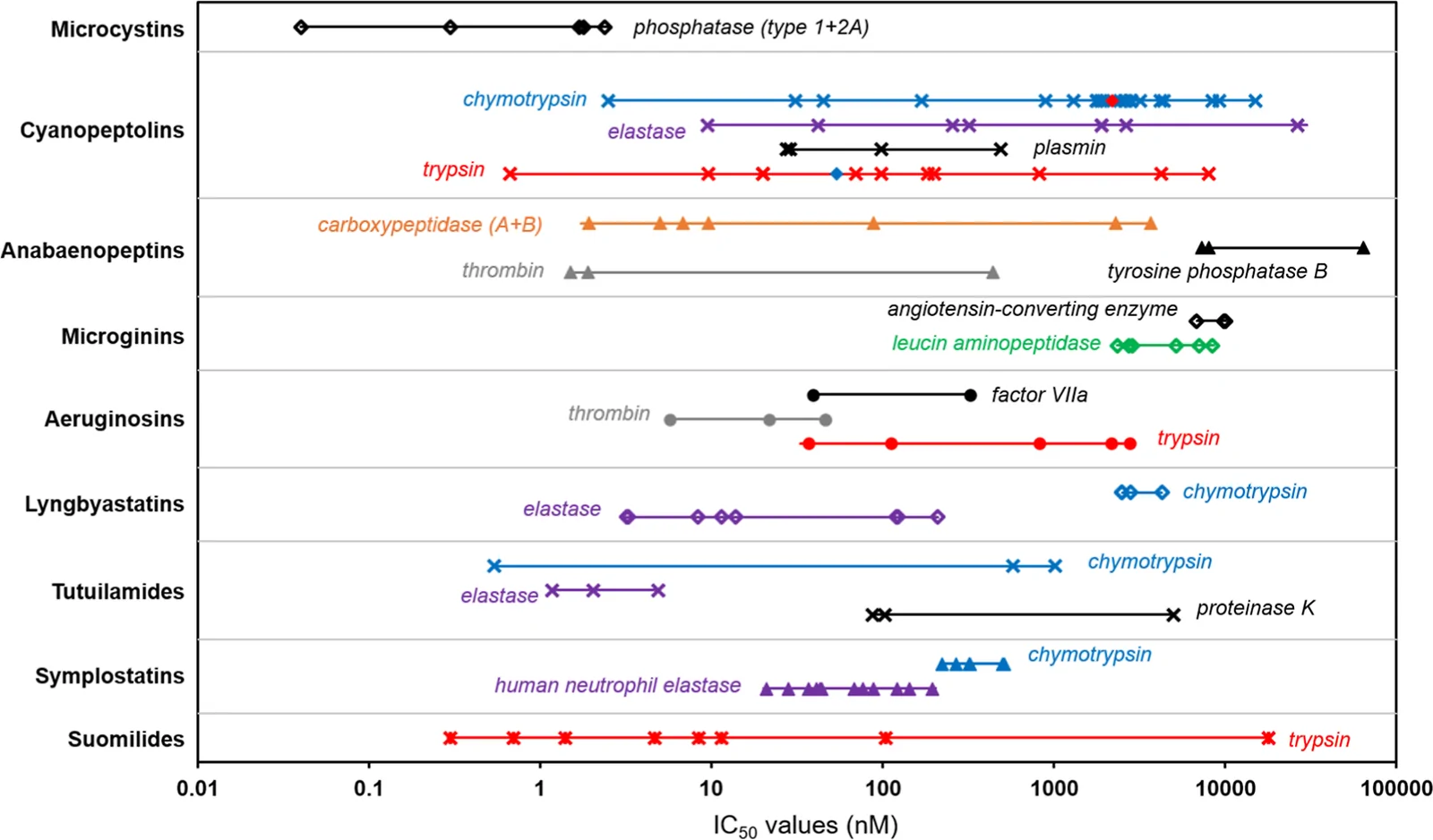
Ranges of inhibitory concentrations of various enzymes (IC_50_ values in nM) by variants from nine cyanopeptide classes. Data points represent values of cyanopeptides in different classes and the *x*-axis is on a logarithmic scale. The enzymes include phosphatase (type 1+2A),^8,45^ chymotrypsin,^14–16,18,46–56^ elastase,^48,50–54,57,58^ plasmin,^14,18,48,59^ trypsin,^14,15,18,47–49,59–66^ carboxypeptidases A and B,^18,48,67,68^ tyrosine phosphatase B,^69^ thrombin,^13,61^ angiotensin-converting enzyme,^18,70^ leucine aminopeptidase,^18,71^ factor VIIa,^61^ and proteinase K.^58^ For cyanopeptolins, the blue diamond for trypsin and red diamond for chymotrypsin represent data obtained in the presented study. Tabulated IC_50_ values are listed in Table S1.

In this study, we evaluated the inhibitory effects of cyanopeptides on aquatic enzymes and three commercial peptidases (trypsin, chymotrypsin, and carboxypeptidase Y). Therefore, cyanobacterial fractions containing mainly cyanopeptolins (fraction M-1), anabaenopeptins (fractions P-1 and P-2), and microginins (fraction M-2) were used. The calculated concentrations of cyanopeptides in each fraction are shown in Table 1. The potential inhibitory effects were generally evaluated by assessing whether the rate of model substrate cleavage decreased after pre-incubating enzymes with cyanopeptides as inhibitor candidates. Because of limited amounts of enzyme material, the potential inhibition effect of cyanopeptides on the oxidase was not evaluated.

### Commercial Peptidases

The fraction from *M. aeruginosa* (M-1 fraction from *mcyD* knocked-out mutant, Table 1) presented inhibition of both trypsin and chymotrypsin, and the measured IC_50_ values of sum cyanopeptolins were 53.75 nM and 2.20 μM, respectively (Figure 5). Inhibition of trypsin and chymotrypsin by cyanopeptolins has previously been reported (Figure 4), and IC_50_ values for trypsin ranged from 0.67 nM^14^ to 8 μM^15^ and for chymotrypsin from 31 nM^16^ to 9.3 μM^52^. Our IC_50_ values fell in the reported ranges. During bloom events, cyanopeptolins have been detected at concentrations similar to these IC_50_ values (e.g., cyanopeptolin 1007 at 14.4 μg/L or 14 nM).^72^ The tested concentrations of trypsin (50 nM) and chymotrypsin (70 nM) are similar, yet cyanopeptolins were 41-fold more potent toward trypsin. Previous studies have reported that the structural diversities of cyanopeptolins at position 2 (e.g., the arginine position connects to 6-hydroxy-2-piperidone (Ahp) for cyanopeptolin A) have significant effects on trypsin and chymotrypsin activities, and the presence of basic amino acid showed a potent inhibitory effect against trypsin, while the presence of hydrophobic residues showed a potent inhibition effect against chymotrypsin.^14,56,73,74^ In this study, the tested cyanopeptolin mixture contained cyanopeptolins A–D, in which the amino acid residues at position 2 are arginine and lysine (modified lysine), which explains the potent inhibition of trypsin. Besides cyanopeptolins, fraction M-1 also contained aerucyclamide A, yet whether this cyanopeptide would inhibit trypsin or chymotrypsin is unknown. One previous study of microcyclamides MZ602 and MZ568, structurally similar to aerucyclamide A, did not detect inhibition of trypsin and only mild inhibition of chymotrypsin by microcyclamide MZ602 (IC_50_ = 75 μM).^75^ Thus, aerucyclamide A is not expected to be the main inhibitor in the M-1 fraction, while cyanopeptolins A, B, C, and D are suspected to explain most of the observed inhibition. The inhibition may also contribute to the stability of cyanopeptolins as substrates during the incubation with trypsin and chymotrypsin, as shown above. Since cyanopeptolins are potent inhibitors of these peptidases, they do not serve as substrates even though their structure would theoretically qualify them as such.

**Figure 5:**
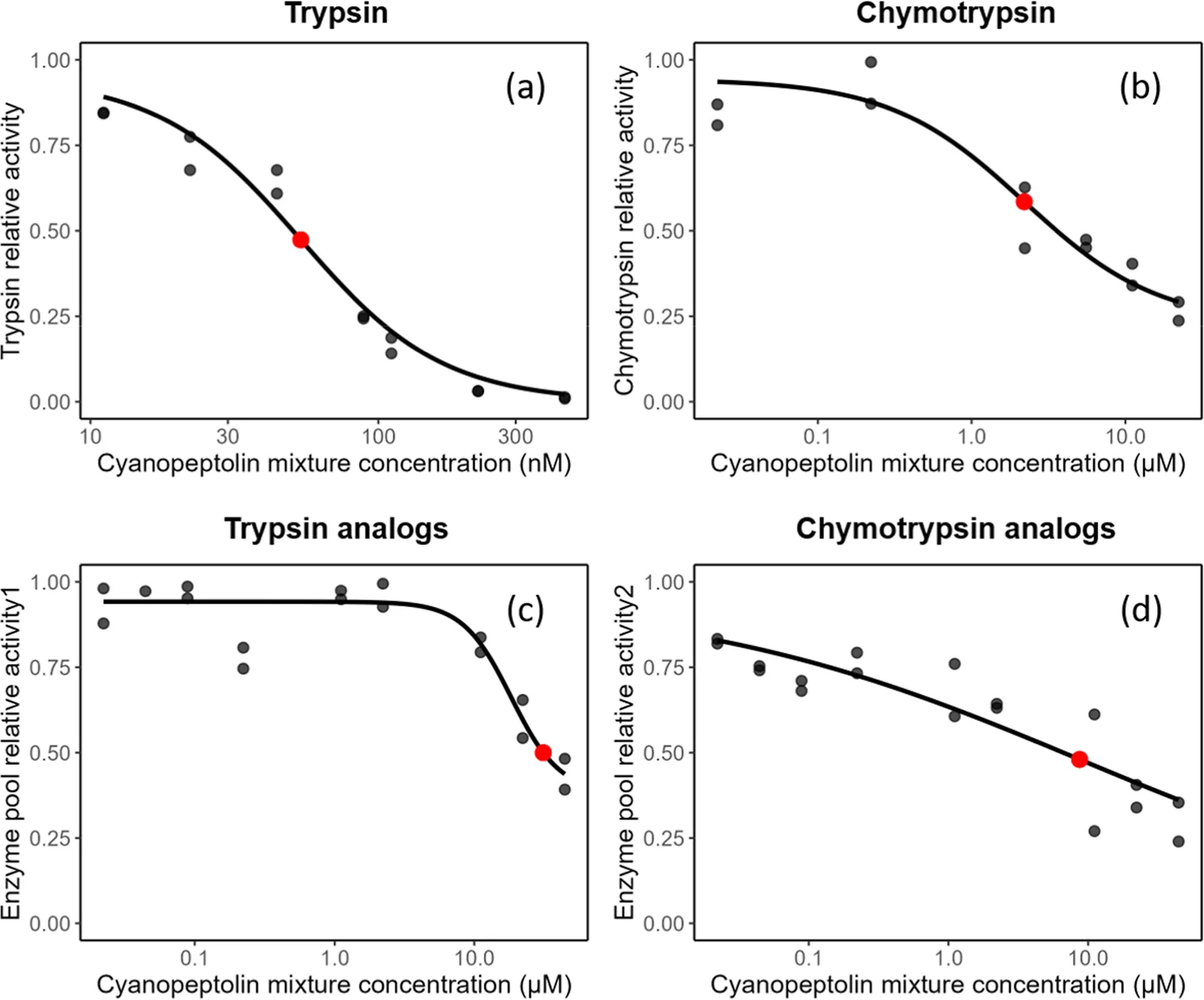
Inhibition by cyanopeptolin-rich M-1 fraction of (a) trypsin, (b) chymotrypsin, and functional analogs in the aquatic enzyme pool tested with (c) the trypsin substrate Z-l-Arg-AMC (activity 1) and (d) the chymotrypsin substrate Ala-Ala-Phe-AMC (activity 2), with marked IC_50_ values (red circle).

The fractions from *P. rubescens* both showed inhibition of trypsin and chymotrypsin up to 100% with fraction P-1 at 2.25 μM sum anabaenopeptins and 56% with fraction P-2 at 1.31 μM sum anabaenopeptins. While anabaenopeptins dominated these fractions, they also contained low quantities of the cyanopeptolin oscillapeptin J with concentrations up to 72.5 nM (P-1) and 12 nM (P-2). As the IC_50_ values obtained from the cyanopeptolin-containing fraction tested before (M-1) are very low, these traces of cyanopeptolins left in the *P. rubescens* fractions are still likely to dominate the observed inhibition. Also, previous studies did not find inhibition by anabaenopeptin A and B^76,77^ but inhibition of trypsin by oscillapeptin J (IC_50_ = 20 nM).^49^ Similar to P-1 and P-2, the microginin-rich fraction M-2 from *M. aeruginosa* (NPCD-01) also showed mild inhibition of trypsin (inhibited by 13%) and chymotrypsin (inhibited by 17%). While the main cyanopeptides were microginins (at 2.56 μM MC-LR equivalent concentration for the sum of microginins), cyanopeptolin 959 was also present at 36 nM (MC-LR equivalent concentration) and is expected to dominate the observed inhibition.

The cyanopeptolin fraction (M-1) did not show inhibition of carboxypeptidase Y at sum cyanopeptolin concentrations of up to 22.2 μM. However, both anabaenopeptin-rich fractions (P-1 and P-2) showed mild inhibition of carboxypeptidase Y. Carboxypeptidase Y activity was inhibited by 5% and 27% when incubated with 9.02 μM sum anabaenopeptins in P-1 and 26.17 μM sum anabaenopeptins in the P-2 fraction, respectively. Based on our other observations, we conclude that the traces of cyanopeptolins in the anabaenopeptin-rich fractions did not contribute significantly to the observed inhibition of carboxypeptidase Y. Microcystins are known as protein phosphatases inhibitors^8,10^ but had not been tested for carboxypeptidase inhibition to date. Previous studies reported inhibition of carboxypeptidase A by anabaenopeptins (Figure 4) with IC_50_ values ranging from low nM to high μM range.^11,18,67,68^ Our results suggested that anabaenopeptins can be mild carboxypeptidase Y inhibitors. The microginin-rich fraction (M-2) showed mild inhibition of carboxypeptidase Y, and the activity reduced to 55% when exposed to 269 μM (sum MC-LR-equivalent) microginins. Again, the minor content of cyanopeptolin 959 is unlikely to be responsible for the observed inhibition. Microginins are reported as inhibitors of leucine aminopeptidase and angiotensin-converting enzyme (Figure 4), yet no studies to date had identified their inhibition of carboxypeptidases, to the best of our knowledge. However, environmentally reported concentrations of anabaenopeptins and microginins are not expected to trigger significant inhibition of carboxypeptidase Y functional analogs.

### Enzyme Pool from the Aquatic Biofilm

To test the inhibition of peptidases in the aquatic enzyme pool, the hydrolysis of added model substrates for peptidases was monitored in the presence of different cyanopeptides. As the reaction rates with model substrates for carboxypeptidase Y (hippuryl-l-Phe and hippuryl-l-Arg) were low, inhibition tests were only conducted for trypsin- and chymotrypsin-like activities, with Z-l-Arg-7-amido-4-AMC and Ala-Ala-Phe-AMC, respectively (Quasi-Michaelis–Menten kinetics in Figure S1). The peptidase activity assessed with both substrates decreased in the presence of cyanopeptolins (fraction M-1), and IC_50_ values of sum cyanopeptolins were assessed as 18.35 μM for trypsin-like and 8.70 μM for chymotrypsin-like activities (Figure 5c,d). The anabaenopeptin fractions (P-1 and P-2) and microginin fraction (M-2) only showed mild inhibition of the peptidase activity in the aquatic enzyme pool. Since cyanopeptolins are potent inhibitors of these peptidases and could not be entirely removed from these fractions, their presence is most likely responsible for the observed mild inhibition. Overall, this study verified that trypsin- and chymotrypsin-like activity in the natural aquatic enzyme pool can be inhibited by cyanopeptolins.

## Implications

Cyanopeptides released from harmful cyanobacterial blooms play dual roles—as enzyme substrates and as enzyme inhibitors. For their role as substrates, we demonstrated that the peptidase activity of the aquatic enzyme pool, separated from the producing organisms, contributed to the transformation of several cyanopeptides. We identified two novel peptide bond hydrolysis products of linear microginins. Carboxypeptidase Y-like activity is likely responsible for microginin degradation in the aquatic enzyme pool as we demonstrated the same reaction and product formation with commercial carboxypeptidase Y. Trypsin, chymotrypsin, and carboxypeptidase Y did not degrade any of the other cyanopeptides tested, including anabaenopeptins, microginins, and cyanopeptolins. The stability in the presence of the peptidases likely results from their cyclic structures, peptide bond modification (e.g., *N*-methylation), and the role of the cyanopeptides as intrinsic inhibitors of these enzymes. The fact that C-terminal amino acids of anabaenopeptins were cleaved in the aquatic enzyme pool points to additional peptidases present in the environment. Oxidation experiments with the multicopper oxidase revealed that, in the presence of the redox mediator ABTS, tyrosine-containing cyanopeptides decayed rapidly, whereas the tyrosine-free congeners remained stable. As the oxidase has prevalent homologs in the environment, including freshwater systems, and natural phenolic or aromatic compounds in dissolved organic matter may act as ABTS-like mediators, oxidation of tyrosine-containing cyanopeptides may represent an important pathway in their environmental fate. The observation of oxidation products in the aquatic enzyme pool suggests that phenolic tyrosine residues are promising targets in cyanopeptide oxidation, which thus may be important for the fate of cyanopeptides in surface waters.

In their role as inhibitors, we demonstrated that cyanopeptolins effectively inhibited trypsin- and chymotrypsin-like activities in the aquatic enzyme pool with IC_50_ values of 18.35 μM and 8.70 μM, respectively, in the range of reported values for commercial enzymes. These inhibitory concentrations have previously been reported during bloom events. On the other hand, anabaenopeptins and microginins showed only mild inhibition of carboxypeptidase Y and little inhibition effect on trypsin, chymotrypsin, and trypsin- and chymotrypsin-like activities in the aquatic enzyme pool. As inhibition of the aquatic enzyme pool was evaluated by model substrates, these results indicated that functional analogs of trypsin, chymotrypsin, and carboxypeptidase Y are present in the environment.

Overall, our findings highlight that cyanopeptides can both inhibit and serve as substrates for aquatic enzymes. Thus, the degradation of cyanopeptides can differ greatly when assessed compound by compound or in mixtures, as they occur in surface water blooms, where inhibitors and substrates coexist. To extrapolate the molecular-scale results presented herein to the field scale, one needs to consider the likelihood that these molecules encounter each other in the environment. While this deserves specific investigations in the future, we already know that cyanopeptides reach relevant concentrations during cyanobacterial blooms and thus hypothesize that they will significantly influence the functioning of aquatic enzymes in the bloom zone. The inhibition of key enzyme functions in the aquatic enzyme pool demonstrates that the biotransformation of other organic substances can be impaired by the presence of cyanopeptides as well, including dissolved organic matter and peptide-based micropollutants. Thus, cyanopeptides may influence the nutrient and carbon turnover rates, as well as metabolic functions of grazing organisms. Consequently, during cyanobacterial bloom events, cyanopeptides can occur in relevant concentrations, hindering their own transformation and that of other relevant organic molecules. Similar processes may be at play in biological water-treatment processes. These insights enhance our understanding of cyanopeptide persistence and transformation in aquatic systems and their role in influencing ecosystem functioning.

## Supplementary Material



## Notes

Additional experiment details, materials, methods, results in Texts S1–S3, Tables S1–S5, and Figures S1–S4 (PDF)
