# Supplementary material for: Dual Roles of Cyanopeptides as Substrates and Inhibitors of Enzymes in Surface Waters
Source: Environ Sci Technol. 2026 Sep 29;60(40):29054–65. doi: 10.1021/acs.est.6c07305 (PMC13674076; doi:10.1021/acs.est.6c07305)
Supplement: Supplementary file 1 [file es6c07305_si_001.pdf]

Dual roles of cyanopeptides as substrates and inhibitors of enzymes in surface waters

Xuejian Wang,[a] Elena Lippuner,[a] Yaochun Yu,[a,b] Elisabeth M.L.- Janssen[a *]

[a] *Swiss Federal Institute of Aquatic Science and Technology (EAWAG), 8600 Dübendorf, Switzerland*

[b]*Department of Civil and Environmental Engineering, Stanford University, 473 Via Ortega, Stanford, California 94305, United States*

**Corresponding author: elisabeth.janssen@eawag.ch*

Supporting Information

The electronic supporting information contains 19 pages including 3 texts, 5 tables, and 4 figures.

List of Texts

Test S1. BCA Protein assay……………………………………………………………………3

Test S2. Cyanobacterial cultures, growth conditions, and extraction…………………………..……3

Test S3. Enzyme activity assay……………………………………………………………………4

List of Tables

Table S1. Enzyme and chemicals…………………………………………………………..…..6

Table S2. Reference standards and bioreagents……………………………………………...6

Table S3. Cyanopeptide list……………………………………………………………..…...8

Table S4. Enzyme reaction experiment details…………………………………………..…..8

Table S5. Literature reported IC50 values……………………………………………...……9

List of Figures

Figure S1. Quasi-Michaelis-Menton kinetics of the aquatic enzyme pool……………………16

Figure S2. Decay kinetics of microginins in the aquatic enzyme pool………………………...17

Figure S3. Structures of tested cyanopeptides……………………………...…………...….18

Figure S4. Highlight of tyrosine residues in structures……………………………..…...….19

Text S1. BCA Protein assay

Pierce™ BCA Protein Assay Kit was used to measure the total protein of the aquatic enzyme pool. 150 µL aquatic enzyme pool was mixed with 150 µL working reagent and incubated at 37 ˚C for 30 min in dark. After 30 min, the plate was cooled down to room temperature in dark. The absorbance at 562 nm was then read by a plate-reader (BioTek, Agilent). A series of BSA standards were also measured to build a standard curve for the quantification of the aquatic enzyme pool.

Text S2. Cyanobacterial cultures, growth conditions, and extraction

Microcystis aeruginosa PCC7806 was originally isolated from Braakman reservoir in The Netherlands (1972) and was obtained from the Pasteur Culture Collection of Cyanobacteria (France). *Planktothrix rubescence* K-0576 was originally isolated from Lake Borre Sø in Denmark, and was obtained from the Norwegian Culture Collection of Algae (NORCCA). *Microcystis aeruginosa* (NPDC-01, isolated from a sewage treatment plant in Cidade de Deus, Rio de Janeiro, Brazil) was obtained from the culture collection of the Laboratory of Ecophysiology and Toxicology of Cyanobacteria, at the Federal University of Rio de Janeiro (Brazil).

NPCD-01 was cultivated in ASM-1 medium (pH 7.5) for 20 days (Gorham et al., 1964), and culture flasks were maintained under a 12 h light/dark photoperiod, with light intensity of 20 µmol photons m-2s-1, continuous aeration, and temperature of 24 ± 2 °C for 20 days. After 20 days of growth, cells were harvested by centrifugation (9000 rpm at 15 °C, 10 min). The resulting biomass was stored at -20 °C, lyophilized (-55 °C, 500 µHg, 24 h, L101 Liotop® lyophilizer, Liobras, Brazil) (Torres et al., 2023). Other cyanobacterial strains were cultured in WC medium at 20 ± 2 °C and irradiated at 12 µmol photons m^{-2} s^{-1} on a 12:12-hlight/dark cycle (Guillard & Lorenzen, 2008). The cells were harvested by centrifugation (rcf of 4000 g at 10 °C, 10 min, Herolab HiCen XL), lyophilized (-40 °C, -3 mbar, 24 h, Lyovac GT2, Leybold) and stored at -20 °C until metabolite extraction.

To extract cyanopeptides, 4 mL of methanol/water (70%/30% v/v) was added to 100 mg of lyophilized cyanobacterial biomass, vortexed, and incubated under sonication (VWR, Ultrasonic cleaner USC-THD, level 6, 15 min at 15 °C). Biomass was separated from

supernatant by centrifugation (*rcf* of 3000 g at 10 °C for 15 min, Megafuge 1.0R), and the supernatant was transferred to a glass bottle. The same extraction was repeated one more time with the same biomass, and the supernatants were combined to yield the biomass extract. The extracts were diluted 30-fold with nanopure water and purified by solid phase extraction (SPE). For the SPE (12-fold vacuum extraction box, Visiprep, 12 ports, Sigma-Aldrich), the SPE cartridge (Oasis HLB 6 cc, 200 mg) was first conditioned with methanol and water (9 mL each) before loading the diluted extracts at a flow rate of 4–5 mL per minute. The cartridge was washed with 9 mL of nanopure water followed by 9 mL of methanol/water (20%/80% v/v) and eluted with 9 mL of methanol/water (85%/15% v/v) at a flow rate of 1–2 mL per minute. The eluted fraction was concentrated to 300 μL by vacuum-assisted evaporation (Syncore Analyst R-12, BÜCHI Labortechnik AG, 40 °C, 120 rpm, 20 mbar) and adjusted gravimetrically to 1.0 mL in ethanol. The final extract was stored at −20 °C until use.

References

1. Gorham, P. R., McLachlan, J., Hammer, U. T., & Kim, W. K. (1964). Isolation and culture of toxic strains of Anabaena flos-aquae (Lyngb.) de Bréb. SIL Proceedings, 1922-2010, 15(2), 796-804. https://doi.org/10.1080/03680770.1962.11895606

2. Torres, M. d. A., Jones, M. R., vom Berg, C., Pinto, E., & Janssen, E. M. L. (2023). Lethal and sublethal effects towards zebrafish larvae of microcystins and other cyanopeptides produced by cyanobacteria. Aquatic Toxicology, 263, 106689. https://doi.org/https://doi.org/10.1016/j.aquatox.2023.106689

3. Guillard, R., & Lorenzen, C. (2008). Yellow-greenalgae with Chlorophyllide. Journal of Phycology, 8, 10-14. https://doi.org/10.1111/j.1529-8817.1972.tb03995.x

Text S3. Enzyme activity assay

Trypsin and chymotrypsin activity were examined by monitoring the fluorescent intensity of 7-amino-4-methylcoumarin (AMC), their shared products when react with their model substrates Z-L-Arg-AMC and Ala-Ala-Phe-AMC, respectively. The tests were conducted by mixing enzyme solutions (50mM ammonium bicarbonate buffer, pH 7.9-8.1) with model substrates under room temperature in transparent 96-well plates (X50 MicroWell Plate, Nunclon Delta, F-bot), the AMC signal was then read immediately at 360/20 nm excitation and 450/20 emission every 6-20 s for 30 min using a plate-reader (BioTek, Agilent). AMC standards

were used to quantify the product concentration. For **carboxypeptidase Y**, the enzyme activity was examined by monitoring the concentration of hippuric acid, the product of hippuryl-L-phenylalanine using an Ultra-high performance liquid chromatography (UHPLC, Dionex UltiMate 3000 RS Pump and RS Autosampler, Thermo Scientific) coupled to a UV/VIS detector (Dionex UltiMate 3000 RS Diode Array Detector, Thermo Scientific). The test was conducted by mixing enzyme solution (50mM ammonium acetate buffer, pH 6.5 adjusted with HCl) with hippuryl-L-phenylalanine under room temperature in a liquid vial and injecting 20 μL of mixed solution every 5 min for 30 min. For the chromatographic separation, a XBridge C18 column (3.5 μm, 2.1 × 50 mm, Waters) was used. The mobile phases were nanopure water (A) and acetonitrile (B) both acidified with formic acid (0.1%). The measurement was run at a flow rate of 350 μL/min at 6.5% of B for 5 min and the column temperature is 40˚C. The detection of hippuric acid was achieved by measuring the UV absorbance at 210 nm as well as comparison of the retention time with a reference standard. To evaluate enzyme inhibition effects by cyanopeptides or model inhibitors, enzyme solutions were mixed and incubated with cyanopeptides or model inhibitors for 40 min before adding model substrates and checking the products intensity. Hippuryl-L-Arg as another potential substrate was tested, however, as the reaction rate was slow, only Hippuryl-L-Phe was used to characterize carboxypeptidase Y activity. As for the aquatic enzyme pool, the abovementioned substrates were tested and corresponding measuring methods were applied.

Table S1. Enzymes and chemicals

	Trypsin assay	Chymotrypsin assay	Carboxypeptidase Y assay
**Enzyme**	trypsin (T8003, Sigma-Aldrich)	α-chymotrypsin (C3991, TCI-Chemical)	carboxypeptidase Y (C3888, Sigma-Aldrich)
**Substrate**	Z-L-Arg-7-amido-4-methylcoumarin hydrochloride (70375-22-3, Cayman Chemical)	Ala-Ala-Phe-7-amido-4-methylcoumarin (A3401, Sigma-Aldrich)	hippuryl-L-phenylalanine (H6875, Sigma-Aldrich); hippuryl-L-arginine (H2508, Sigma-Aldrich)
**Buffer**	ammonium bicarbonate (A6141, Sigma-Aldrich), pH7.9-8.1	ammonium bicarbonate (A6141, Sigma-Aldrich), pH7.9-8.1	ammonium acetate (Sigma, Aldrich), pH6.5

Table S2. Reference standards and bioreagents used in the metabolite analysis

Reference standards and bioreagents used in the metabolite analysis with their molecular formula, monoisotopic mass, and dominant precursor ion form with associated mass-to-charge (m/z) ratio.

metabolite	molecular formula	Monoisotopic mass (Da; rounded to 5 d.p.)	dominant precursor ion and *m/z* value	
Aerucyclamide A	$C_{24}H_{34}N_6O_4S_2$	534.20830	[M&H][&]	535.21557
Anabaenopeptin A	$C_{44}H_{57}N_7O_{10}$	843.41669	[M&H] [&]	844.42397
Anabaenopeptin B	$C_{41}H_{60}N_{10}O_9$	836.45447	[M&H] [&]	837.46175
Oscillamide Y	$C_{45}H_{59}N_7O_{10}$	857.43234	[M&H] [&]	858.43962
Cyanopeptolin A	$C_{46}H_{72}N_{10}O_{12}$	956.53312	[M&H] [&]	957.54039
Cyanopeptolin D	$C_{48}H_{76}N_8O_{12}$	956.55827	[M&H] [&]	957.56555
MC-LR	$C_{49}H_{74}N_{10}O_{12}$	994.54877	[M&H] [&]	995.55604
MC-HilR	$C_{50}H_{76}N_{10}O_{12}$	1008.56442	[M&H] [&]	1009.57169
MC-LA	$C_{46}H_{67}N_7O_{12}$	909.48477	[M&H] [&]	910.49205
MC-LF	$C_{52}H_{71}N_7O_{12}$	985.51607	[M&H] [&]	986.52335

MC-LW	$C_{54}H_{72}N_8O_{12}$	1024.52697	$[M\&H]^{\&}$	1025.53425
MC-LY	$C_{52}H_{71}N_7O_{13}$	1001.51099	$[M\&H]^{\&}$	1002.51826
MC-RR	$C_{49}H_{75}N_{13}O_{12}$	1037.56581	$[M\&2H]^{2\&}$	519.79018
MC-YR	$C_{52}H_{72}N_{10}O_{13}$	1044.52803	$[M\&H]^{\&}$	1045.53531
[D-Asp^3,(*E*)-Dhb^7]MC-RR	$C_{48}H_{73}N_{13}O_{12}$	1023.55016	$[M\&2H]^{2\&}$	512.78236
[D-Asp^3]MC-LR	$C_{48}H_{72}N_{10}O_{12}$	980.53312	$[M\&H]^{\&}$	981.54039
Nodularin-R	$C_{41}H_{60}N_8O_{10}$	824.44324	$[M\&H]^{\&}$	825.45052
Aeruginosin 98B	$C_{29}H_{46}N_6O_9S$	654.30470	$[M\&H]^{\&}$	655.31197

Other Materials. Microcystin reference standards MC-LR, MC-YR, MC-RR, MC-LF, MC-LA, MC-LW, MC-LY, and nodularin (all >95% purity by HPLC) were obtained from Enzo Life Science (Lausen, Switzerland) and [D-Asp^3, E-Dhb^7] MC-RR (>95% purity by HPLC) from CyanoBiotech GmbH (Berlin, Germany). Bioreagents for aeruginosin 98B, cyanopeptolin A, cyanopeptolin D, anabaenopeptin A, anabaenopeptin B, and oscillamide Y (all >90% purity by HPLC) were obtained from CyanoBiotech. Aerucyclamide A was obtained as purified bioreagent in dimethyl sulfoxide by Prof. Karl Gademann (University Zurich, Switzerland) (Portmann et al., 2008).

Reference

Portmann, C., Blom, J. F., Gademann, K., & Jüttner, F. (2008). Aerucyclamides A and B: Isolation and Synthesis of Toxic Ribosomal Heterocyclic Peptides from the Cyanobacterium Microcystis aeruginosa PCC 7806. Journal of Natural Products, 71(7), 1193-1196. https://doi.org/10.1021/np800118g

Table S3. Cyanopeptide list for the study

Microcystin	Molecular formula	Confidence level	Source
MC-LR	$C_{49}H_{74}N_{10}O_{12}$	level 1	*M. aeruginosa* PCC7806 and purchased standard
[D-Asp3]MC-LR	$C_{48}H_{72}N_{10}O_{12}$	level 1	*M. aeruginosa* PCC7806
MC-YR	$C_{52}H_{72}N_{10}O_{13}$	level 1	purchased standard
MC-LY	$C_{52}H_{71}N_7O_{13}$	level 1	purchased standard
[D-Asp3,(E)-Dhb7]MC-RR	$C_{48}H_{73}N_{13}O_{12}$	level 1	*P. rubescens* (K-0576)
**Anabaenopeptin**			
Anabaenopeptin A	$C_{44}H_{57}N_7O_{10}$	level 1	*P. rubescens* (K-0576)
Anabaenopeptin B	$C_{41}H_{60}N_{10}O_9$	level 1	*P. rubescens* (K-0576)
Oscillamide Y	$C_{45}H_{59}N_7O_{10}$	level 1	*P. rubescens* (K-0576)
Anabaenopeptin F	$C_{42}H_{62}N_{10}O_9$	level 2b	*P. rubescens* (K-0576)
**Cyanopeptolin**			
Cyanopeptolin A	$C_{46}H_{72}N_{10}O_{12}$	level 1	*M. aeruginosa* (*mcyD* knocked-out mutant)
Cyanopeptolin B	$C_{46}H_{71}N_8O_{12}H$	level 2b	*M. aeruginosa* (*mcyD* knocked-out mutant)
Cyanopeptolin C	$C_{47}H_{74}N_8O_{12}$	level 2b	*M. aeruginosa* (*mcyD* knocked-out mutant)
Cyanopeptolin D	$C_{48}H_{76}N_8O_{12}$	level 1	*M. aeruginosa* (*mcyD* knocked-out mutant)
Oscillapeptin J	$C_{47}H_{68}N_{10}O_{18}S$	level 2b	*P. rubescens* (K-0576)
Cyanopeptolin 959	$C_{43}H_{61}N_9O_{14}S$	level 2b	*M. aeruginosa* (NPDC-01)
**Microginin**			
Nostoginin BN741	$C_{39}H_{59}N_5O_9$	level 2b	*M. aeruginosa* (NPDC-01)
Microginin FR6	$C_{39}H_{57}N_5O_9$	level 2b	*M. aeruginosa* (NPDC-01)
**others**			
Aerucyclamide A	$C_{24}H_{34}N_6O_4S_2$	level 1	*M. aeruginosa* (*mcyD* knocked-out mutant)
Planktocyclin	$C_{39}H_{60}N_8O_8S$	level 2b	*P. rubescens* (K-0576)

Table S4. Enzyme reaction experiment details

Enzyme	Cyanopeptides	Experiment
Trypsin (5μM)	Fractions: (1)M-1 from *M. aeruginosa* (*mcy*D knocked out); (2) P-1 from *P. rubescens*	Fractions were spiked in trypsin solution and incubated at room temperature, sub-sampling were conducted at 0d, 6h, 1d, 2d and 4d.
Chymotrypsin (10 units/mL)	Fraction: (1) M-1 form *M. aeruginosa* (*mcy*D knocked out); (2) P-1 and P-2 from *P.*	Fractions were spiked in chymotrypsin solution and incubated at room temperature, sub-sampling were conducted at 0d, 6h, 1d, 2d and 4d.

	rubescens; (3)M-2 from *M. aeruginosa* NPDC-01	

Carboxypeptidase Y (1 unit/mL)	Fractions: (1) P-1 and P-2 from *P. rubescens*; (2) M-2 from *M. aeruginosa* NPDC-01	Fractions were spiked in carboxypeptidase Y solution and incubated at room temperature, sub-sampling were conducted at 0d, 6h, 1d, 2d and 4d.
Oxidase	Fractions: P-1 and P-2 from *P. rubescens*.<br>Extract from: (1) *M. aeruginosa* (NPDC-01); (2) *P. rubescens;* (3) *M. aeruginosa* PCC7806<br>Standards: MC-LR, MC-LY and MC-YR	Fractions, extracts and standards were spiked in MCO1 solution with/without ABTS or only ABTS solution; and incubated at 30 ˚C, sub-sampling was conducted at 0d, (6/8h), 1d (2d).
Aquatic enzyme pool	Extract from: (1) *M. aeruginosa* NPDC-01; (2) *P. rubescens;* (3) *M. aeruginosa* PCC7806	Fractions or extracts were spiked in biofilm enzyme pool and incubated at room temperature, sub-sampling were conducted at 0d, 6h, 1d, 2d and 4d.

Table S5. Literature reported IC_{50} values of cyanopeptides toward different enzymes (S5-1). Note for trypsin and chymotrypsin, the used enzyme concentrations were also included in S5-2.

Table S5-1

Cyanopeptide class	Cyanopeptide	Value	Units	Tested enzyme	Reference
Microcystin	MC-LR	1.7	nM	phosphatase PP1	Honkanen & Zwiller 1990
Microcystin	MC-LR	0.3	nM	phosphatase PP1	An & Carmichael 1994
Microcystin	[D-Asp3 ,(E)-Dhb7]MC-RR	1.8	nM	phosphatase PP1	Blom 2005
Microcystin	[D-Asp3 ,(E)-Dhb7]MC-RR	2.4	nM	phosphatase PP2A	Blom 2005
Microcystin	MC-LR	0.04	nM	phosphatase PP2A	Honkanen & Zwiller 1990
Cyanopeptolin	Cyanopeptolin 1020	1.8	µM	chymotrypsin	Gademann & Portmann 2010
Cyanopeptolin	Micropeptin T1	3	µg/mL	chymotrypsin	Kodani & Suzuki 1999
Cyanopeptolin	Cyanopeptolin 963A	0.9	µM	chymotrypsin	Bister & Keller 2004

Cyanopeptolin	Micropeptin SF909	4	µg/ mL	chymotrypsin	Banker & Carmeli1999
Cyanopeptolin	Cyanopeptolin 954	45	nM	chymotrypsin	Von Elert & Oberer 2005
Cyanopeptolin	Nostopeptin BN920	31	nM	chymotrypsin	Von Elert & Oberer 2005
Cyanopeptolin	Micropeptin SD979	2.4	µg/ mL	chymotrypsin	Reshef Tetrahedron 2001
Cyanopeptolin	Micropeptin SD1002	3.2	µg/ mL	chymotrypsin	Reshef Tetrahedron 2001
Cyanopeptolin	Oscillapeptin A	2.2	µg/ mL	chymotrypsin	Ituo & Ishida 1999
Cyanopeptolin	Oscillapeptin B	2.1	µg/ mL	chymotrypsin	Ituo & Ishida 1999
Cyanopeptolin	Oscillapeptin C	3	µg/ mL	chymotrypsin	Ituo & Ishida 1999
Cyanopeptolin	Oscillapeptin D	2.2	µg/ mL	chymotrypsin	Ituo & Ishida 1999
Cyanopeptolin	Oscillapeptin E	3	µg/ mL	chymotrypsin	Ituo & Ishida 1999
Cyanopeptolin	Oscillapeptin J	2.8	µM	chymotrypsin	Blom 2006
Cyanopeptolin	Somamide B	4.2	µM	chymotrypsin	Taori 2007
Cyanopeptolin	Kempopeptin A	2.6	µM	chymotrypsin	Taori 2008
Cyanopeptolin	Kempopeptin B	8.4	µM	chymotrypsin	Taori 2008
Cyanopeptolin	Bouillomide A	0.17	µM	bovine pancrease a-chymotrypsin	Rubio 2010
Cyanopeptolin	Bouillomide B	9.3	µM	bovine pancrease a-chymotrypsin	Rubio 2010
Cyanopeptolin	Micropeptin 88-N	15	µM	α-chymotrypsin type II	Yamaki 2004
Cyanopeptolin	Micropeptin 88-Y	1.3	µM	α-chymotrypsin type II	Yamaki 2004
Cyanopeptolin	Micropeptin T-20	0.00 3	µM	chymotrypsin	Okano 1999
Cyanopeptolin	Oscillapeptin A	0.3	µg/ mL	elastase	Ituo & Ishida 1999
Cyanopeptolin	Oscillapeptin B	0.05	µg/ mL	elastase	Ituo & Ishida 1999
Cyanopeptolin	Oscillapeptin D	30	µg/ mL	elastase	Ituo & Ishida 1999
Cyanopeptolin	Oscillapeptin E	3	µg/ mL	elastase	Ituo & Ishida 1999
Cyanopeptolin	Somamide B	9.5	nM	elastase	Taori 2007
Cyanopeptolin	Kempopeptin A	0.32	µM	elastase	Taori 2008
Cyanopeptolin	Bouillomide A	1.9	µM	elastase	Rubio 2010
Cyanopeptolin	Bouillomide B	1.9	µM	elastase	Rubio 2010
Cyanopeptolin	Cyanopeptolin 1020	3.9	nM	Factor Xia	Gademann & Portmann 2010
Cyanopeptolin	Cyanopeptolin 1020	4.5	nM	human kallikrein	Gademann & Portmann 2010
Cyanopeptolin	Cyanopeptolin 1020	0.49	µM	plasmin	Gademann & Portmann 2010
Cyanopeptolin	Micropeptin T2	0.1	µg/ mL	plasmin	Kodani & Suzuki 1999
Cyanopeptolin	Micropeptin A90720A	30	ng/ mL	plasmin	Bonjouklian+Smitka 1996
Cyanopeptolin	Oscillapeptin F	0.03	µg/ mL	plasmin	Ituo & Ishida 1999
Cyanopeptolin	Micropeptin A90720A	270	ng/ mL	thrombin	Bonjouklian & Smitka 1996
Cyanopeptolin	Cyanopeptolin 1020	0.67	nM	trypsin	Gademann & Portmann 2010
Cyanopeptolin	Micropeptin A90720A	10	ng/ mL	trypsin	Bonjouklian & Smitka 1996
Cyanopeptolin	Micropeptin SF995	0.2	µg/ mL	trypsin	Banker & Carmeli1999

Cyanopeptolin	Micropeptin SF608	0.5	µg/mL	trypsin	Banker & Carmeli1999
Cyanopeptolin	Micropeptin SD999	8	µg/mL	trypsin	Reshef Tetrahedron 2001
Cyanopeptolin	Micropeptin SD944	4	µg/mL	trypsin	Reshef Tetrahedron 2001
Cyanopeptolin	Oscillapeptin F	0.2	µg/mL	trypsin	Ituo & Ishida 1999
Cyanopeptolin	Oscillapeptin J	20	nM	trypsin	Blom 2006
Cyanopeptolin	Micropeptin T2	0.1	µg/mL	trypsin	Kodani & Suzuki 1999
Cyanopeptolin	Micropeptin 90	0.02	µM	trypsin	Ishida 1995
Cyanopeptolin	Micropeptin A	0.07	µM	trypsin	Okino 1993
Anabaenopeptin	Brunsvicamide A	5	nM	carboxypeptidase A	Walther & Renner 2009
Anabaenopeptin	Anabaenopeptin G	0.002	µg/mL	carboxypeptidase A	Ituo & Ishida 1999
Anabaenopeptin	Anabaenopeptin H	3.4	µg/mL	carboxypeptidase A	Ituo & Ishida 1999
Anabaenopeptin	Anabaenopeptin I	5.2	ng/mL	carboxypeptidase A	Murakami 2000
Anabaenopeptin	Anabaenopeptin J	7.6	ng/mL	carboxypeptidase A	Murakami 2000
Anabaenopeptin	Anabaenopeptin T	2	µg/mL	carboxypeptidase A	Kodani & Suzuki 1999
Anabaenopeptin	Brunsvicamide A	88	nM	carboxypeptidase B	Walther & Renner 2009
Anabaenopeptin	Oscillamide Y	10	µM	chymotrypsin	Sano & Kaya 1995
Anabaenopeptin	Brunsvicamide A	64.2	µM	Mycobacterium tuberculosis tyrosine phosphatase B	Muller & Krick 2006
Anabaenopeptin	Brunsvicamide B	7.3	µM	Mycobacterium tuberculosis tyrosine phosphatase B	Muller & Krick 2006
Anabaenopeptin	Brunsvicamide C	8	µM	Mycobacterium tuberculosis tyrosine phosphatase B	Muller & Krick 2006
Anabaenopeptin	Anabaenopeptin A	440	nM	thrombin	Schreuder 2016
Anabaenopeptin	Anabaenopeptin B	1.5	nM	thrombin	Schreuder 2016
Anabaenopeptin	Anabaenopeptin C	1.9	nM	thrombin	Schreuder 2016
Anabaenopeptin	Schizopeptin 791	45	µg/mL	trypsin	Reshef & Carmeli, 2002
Microginin	Microginin 51A	4.5	µg/mL	aminopeptidase M	Ishida & Kato 2000
Microginin	Microginin T1	5	µg/mL	angiotensin-converting enzyme	Kodani & Suzuki 1999
Microginin	Microginin T2	7	µg/mL	angiotensin-converting enzyme	Kodani & Suzuki 1999
Microginin	Microginin	7	µg/mL	angiotensin-converting enzyme	Okino & Matsuda 1993
Microginin	Microginins 299-C	2	µg/mL	leucin aminopeptidase	Ishida & Matsuda1998
Microginin	Microginins 299-D	6.4	µg/mL	leucin aminopeptidase	Ishida & Matsuda1998
Microginin	Microginins 299-A	4.6	µg/mL	leucine aminopeptidase	Ishida & Matsuda 1997
Microginin	Microginins 299-B	6.5	µg/mL	leucine aminopeptidase	Ishida & Matsuda 1997
Microginin	Microginin T1	2	µg/mL	leucine aminopeptidase	Kodani & Suzuki 1999
Microginin	Microginin T2	2	µg/mL	leucine aminopeptidase	Kodani & Suzuki 1999
Aeruginosin	Chlorodysinosin A	39	nM	factor VIIa	Hanessian 2006
Aeruginosin	Dysinosin A	326	nM	factor VIIa	Hanessian 2006

Aeruginosin	Aeruginosin 828A	21.8	nM	thrombin	Kohler & Grundler 2014
Aeruginosin	Chlorodysinosin A	5.7	nM	thrombin	Hanessian 2006
Aeruginosin	Chlorodysinosin A	46	nM	thrombin	Hanessian 2006
Aeruginosin	Dysinosin A	46	nM	thrombin	Hanessian 2006
Aeruginosin	Aeruginosin 828A	112	nM	trypsin	Kohler & Grundler 2014
Aeruginosin	Chlorodysinosin A	37	nM	trypsin	Hanessian 2006
Aeruginosin	Aeruginosin KY642	1.4	µg/ mL	trypsin	RavehCarmeli 2009
Aeruginosin	Aeruginosin KY608	1.7	µg/ mL	trypsin	RavehCarmeli 2009
Aeruginosin	MicrocinSF608	0.5	µg/ mL	trypsin	Banker 1999
Lyngbyastatin	Lyngbyastatin 4	4.3	µM	67 ug/mL	Matthew 2007
Lyngbyastatin	Lyngbyastatin 5	2.8	µM	67 ug/mL	Taori 2007
Lyngbyastatin	Lyngbyastatin 6	2.5	µM	67 ug/mL	Taori 2007
Lyngbyastatin	Lyngbyastatin 7	2.5	µM	67 ug/mL	Taori 2007
Lyngbyastatin	Lyngbyastatin 4	13.9	nM	3.75 ug/mL	Matthew 2007
Lyngbyastatin	Lyngbyastatin 5	3.2	nM	3.75 ug/mL	Taori 2007
Lyngbyastatin	Lyngbyastatin 6	3.3	nM	3.75 ug/mL	Taori 2007
Lyngbyastatin	Lyngbyastatin 7	8.3	nM	3.75 ug/mL	Taori 2007
Lyngbyastatin	Lyngbyastatin 7	11.5	nM	3.75 ug/mL	Taori 2007
Lyngbyastatin	Lyngbyastatin 8	123	nM	3.75 ug/mL	Kwan 2009
Lyngbyastatin	Lyngbyastatin 9	210	nM	3.75 ug/mL	Kwan 2009
Lyngbyastatin	Lyngbyastatin 10	120	nM	3.75 ug/mL	Kwan 2009
Tutuilamide	Tutuilamide A	1.014	µM	huamn chymotrypsin	Keller 2020
Tutuilamide	Tutuilamide B	0.577	µM	huamn chymotrypsin	Keller 2020
Tutuilamide	Tutuilamide C	0.542	µM	huamn chymotrypsin	Keller 2020
Tutuilamide	Tutuilamide A	1.18	nM	pancreatic elastase	Keller 2020
Tutuilamide	Tutuilamide B	2.05	nM	pancreatic elastase	Keller 2020
Tutuilamide	Tutuilamide C	4.93	nM	pancreatic elastase	Keller 2020
Tutuilamide	Tutuilamide A	103.7	nM	proteinase K	Keller 2020
Tutuilamide	Tutuilamide B	87.6	nM	proteinase K	Keller 2020
Tutuilamide	Tutuilamide C	5000	nM	proteinase K	Keller 2020
Symplostatin	Symplostatin 5	322	nM	bovine pancreatic chymotrypsin	Salvador 2013
Symplostatin	Symplostatin 6	503	nM	bovine pancreatic chymotrypsin	Salvador 2013
Symplostatin	Symplostatin 7	515	nM	bovine pancreatic chymotrypsin	Salvador 2013
Symplostatin	Symplostatin 8	268	nM	bovine pancreatic chymotrypsin	Salvador 2013
Symplostatin	Symplostatin 9	324	nM	bovine pancreatic chymotrypsin	Salvador 2013
Symplostatin	Symplostatin 10	222	nM	bovine pancreatic chymotrypsin	Salvador 2013
Symplostatin	Symplostatin 2	5.41	nM	elastase	Keller 2020
Symplostatin	Symplostatin 5	144	nM	human neutrophil elastase	Salvador 2013
Symplostatin	Symplostatin 6	121	nM	human neutrophil elastase	Salvador 2013
Symplostatin	Symplostatin 7	195	nM	human neutrophil elastase	Salvador 2013
Symplostatin	Symplostatin 8	41	nM	human neutrophil elastase	Salvador 2013
Symplostatin	Symplostatin 9	28	nM	human neutrophil elastase	Salvador 2013
Symplostatin	Symplostatin 10	21	nM	human neutrophil elastase	Salvador 2013

Symplostatin	Symplostatin 5	68	nM	porcine pancreatic elastase	Salvador 2013
Symplostatin	Symplostatin 6	89	nM	porcine pancreatic elastase	Salvador 2013
Symplostatin	Symplostatin 7	77	nM	porcine pancreatic elastase	Salvador 2013
Symplostatin	Symplostatin 8	43	nM	porcine pancreatic elastase	Salvador 2013
Symplostatin	Symplostatin 9	37	nM	porcine pancreatic elastase	Salvador 2013
Symplostatin	Symplostatin 10	44	nM	porcine pancreatic elastase	Salvador 2013
suomilide	suomilide G	0.7	nM	trypsin	Naimi 2026
suomilide	suomilide H	1.4	nM	trypsin	Naimi 2026
suomilide	suomilide B	0.3	nM	trypsin	Naimi 2026
suomilide	suomilide D	8.4	nM	trypsin	Naimi 2026
suomilide	suomilide	104	nM	human trypsin-1	Ahmed 2021
suomilide	suomilide	4.7	nM	human trypsin-2	Ahmed 2021
suomilide	suomilide	11.5	nM	human trypsin-3	Ahmed 2021
suomilide	suomilide	18	µM	non-human trypsin isoenzyme	Fujii 1997
suomilide	Suomilides	7.6	nM	humn plasmin	Ahmed 2021
suomilide	suomilide	85	nM	plasma kallikrein	Ahmed 2021
suomilide	suomilide	114	nM	Factor Xa	Ahmed 2021
suomilide	suomilide	71	µM	huamn urokinase	Ahmed 2021
suomilide	suomilide	100	µM	human thrombin	Ahmed 2021

Table S5-2

Cyanopeptide class	Cyanopeptide	IC50 value/ nM	Tested enzyme or species	enzyme concentration	Reference
Cyanopeptolin	Cyanopeptolin 1020	1	trypsin	0.1 nM	Gademann, Portmann 2010
Cyanopeptolin	Micropeptin A90720A	10	trypsin	/	Bonjouklian+Smitka 1996
Cyanopeptolin	Micropeptin SF995	201	trypsin	45 µg/mL	Banker,Carmeli1999
Cyanopeptolin	Micropeptin SF608	821	trypsin	45 µg/mL	Banker,Carmeli1999
Cyanopeptolin	Micropeptin SD999	7999	trypsin	45 µg/mL	Reshef Tetrahedron 2001
Cyanopeptolin	Micropeptin SD944	4232	trypsin	45 µg/mL	Reshef Tetrahedron 2001
Cyanopeptolin	Oscillapeptin F	184	trypsin	/	Ituo,Ishida 1999
Cyanopeptolin	Oscillapeptin J	20	Trypsin	0.051 U/200µl	Blom 2006
Cyanopeptolin	Micropeptin T2	98	tyrosin	/	Kodani,Suzuki 1999
Cyanopeptolin	Micropeptin 90	20	tyrosin	/	Ishida 1995
Cyanopeptolin	Micropeptin A	70	tyrosin	/	Okino 1993
Anabaenopeptin	Schizopeptin 791	56819	trypsin	45 µg/mL	Reshef+ Carmeli, 2002
Aeruginosin	Chlorodysinosin A	37	trypsin	/	Hanessian, Del Valle et al. 2006
Aeruginosin	Aeruginosin KY642	2175	trypsin	45 µg/mL	RavehCarmeli_2009
Aeruginosin	Aeruginosin KY608	2791	trypsin	45 µg/mL	RavehCarmeli_2009
Aeruginosin	MicrocinSF608	821	trypsin	45 µg/mL	Banker 1999
Suomilide	suomilide G	1	trypsin	100 pM	Naimi 2026
Suomilide	suomilide H	1	trypsin	100 pM	Naimi 2026
Suomilide	suomilide B	0	trypsin	100 pM	Naimi 2026

Suomilide	suomilide D	8	trypsin	100 pM	Naimi 2026
Suomilide	suomilide	104	human trypsin-1	1.88 nM	Ahmed 2021
Suomilide	suomilide	5	human trypsin-2	1.88 nM	Ahmed 2021
Suomilide	suomilide	12	human trypsin-3	1.88 nM	Ahmed 2021
Suomilide	suomilide	18000	non-human trypsin isoenzyme	/	Fujii 1997
Cyanopeptolin	Cyanopeptolin 1020	1800	chymotrypsin	0.051 U / 200 uL	Gademann, Portmann 2010
Cyanopeptolin	Micropeptin T1	2854	chymotrypsin	/	Kodani,Suzuki 1999
Cyanopeptolin	Cyanopeptolin 963A	900	chymotrypsin	7.7 U/mL	Bister, Keller 2004
Cyanopeptolin	Micropeptin SF909	4395	chymotrypsin	45 µg/mL	Banker,Carmeli1999
Cyanopeptolin	Cyanopeptolin 954	45	chymotrypsin	/	Von Elert+Oberer 2005
Cyanopeptolin	Nostopeptin BN920	31	chymotrypsin	/	Von Elert+Oberer 2005
Cyanopeptolin	Micropeptin SD979	2449	chymotrypsin	45 µg/mL	Reshef Tetrahedron 2001
Cyanopeptolin	Micropeptin SD1002	3190	chymotrypsin	45 µg/mL	Reshef Tetrahedron 2001
Cyanopeptolin	Oscillapeptin A	1883	chymotrypsin	/	Ituo,Ishida 1999
Cyanopeptolin	Oscillapeptin B	1776	chymotrypsin	/	Ituo,Ishida 1999
Cyanopeptolin	Oscillapeptin C	2824	chymotrypsin	/	Ituo,Ishida 1999
Cyanopeptolin	Oscillapeptin D	1950	chymotrypsin	/	Ituo,Ishida 1999
Cyanopeptolin	Oscillapeptin E	2636	chymotrypsin	/	Ituo,Ishida 1999
Cyanopeptolin	Oscillapeptin J	2800	chymotrypsin	0.04 U/200 µl	Blom 2006
Cyanopeptolin	Somamide B	4200	chymotrypsin	67 µg/mL	Taori 2007
Cyanopeptolin	Kempopeptin A	2600	chymotrypsin	67 µg/mL	Taori 2008
Cyanopeptolin	Kempopeptin B	8400	chymotrypsin	67 µg/mL	Taori 2008
Cyanopeptolin	Bouillomide A	170	bovine pancrease a-chymotrypsin	10 µg/mL	Rubio 2010
Cyanopeptolin	Bouillomide B	9300	bovine pancrease a-chymotrypsin	10 µg/mL	Rubio 2010
Cyanopeptolin	Micropeptin 88-N	15000	α-chymotrypsin type II	/	Yamaki 2004
Cyanopeptolin	Micropeptin 88-Y	1300	α-chymotrypsin type II	/	Yamaki 2004
Cyanopeptolin	Micropeptin T-20	3	chymotrypsin	/	Okano 1999
Lyngbyastatin	Lyngbyastatin 4	4300	bovine pancreas α-chymotrypsin	67 µg/mL	Matthew 2007
Lyngbyastatin	Lyngbyastatin 5	2800	bovine pancreas α-chymotrypsin	67 µg/mL	Taori 2007
Lyngbyastatin	Lyngbyastatin 6	2500	bovine pancreas α-chymotrypsin	67 µg/mL	Taori 2007
Lyngbyastatin	Lyngbyastatin 7	2500	bovine pancreas α-chymotrypsin	67 µg/mL	Taori 2007
Tutuilamide	Tutuilamide A	1014	huamn chymotrypsin	10 nM	Keller 2020
Tutuilamide	Tutuilamide B	577	huamn chymotrypsin	10 nM	Keller 2020
Tutuilamide	Tutuilamide C	1	huamn chymotrypsin	10 nM	Keller 2020
Symplostatin	Symplostatin 5	322	bovine pancreatic chymotrypsin	5 µg/mL	Salvador 2013
Symplostatin	Symplostatin 6	503	bovine pancreatic chymotrypsin	5 µg/mL	Salvador 2013
Symplostatin	Symplostatin 7	515	bovine pancreatic chymotrypsin	5 µg/mL	Salvador 2013
Symplostatin	Symplostatin 8	268	bovine pancreatic chymotrypsin	5 µg/mL	Salvador 2013

Symplostatin	Symplostatin 9	324	bovine pancreatic chymotrypsin	5 µg/mL	Salvador 2013
Symplostatin	Symplostatin 10	222	bovine pancreatic chymotrypsin	5 µg/mL	Salvador 2013

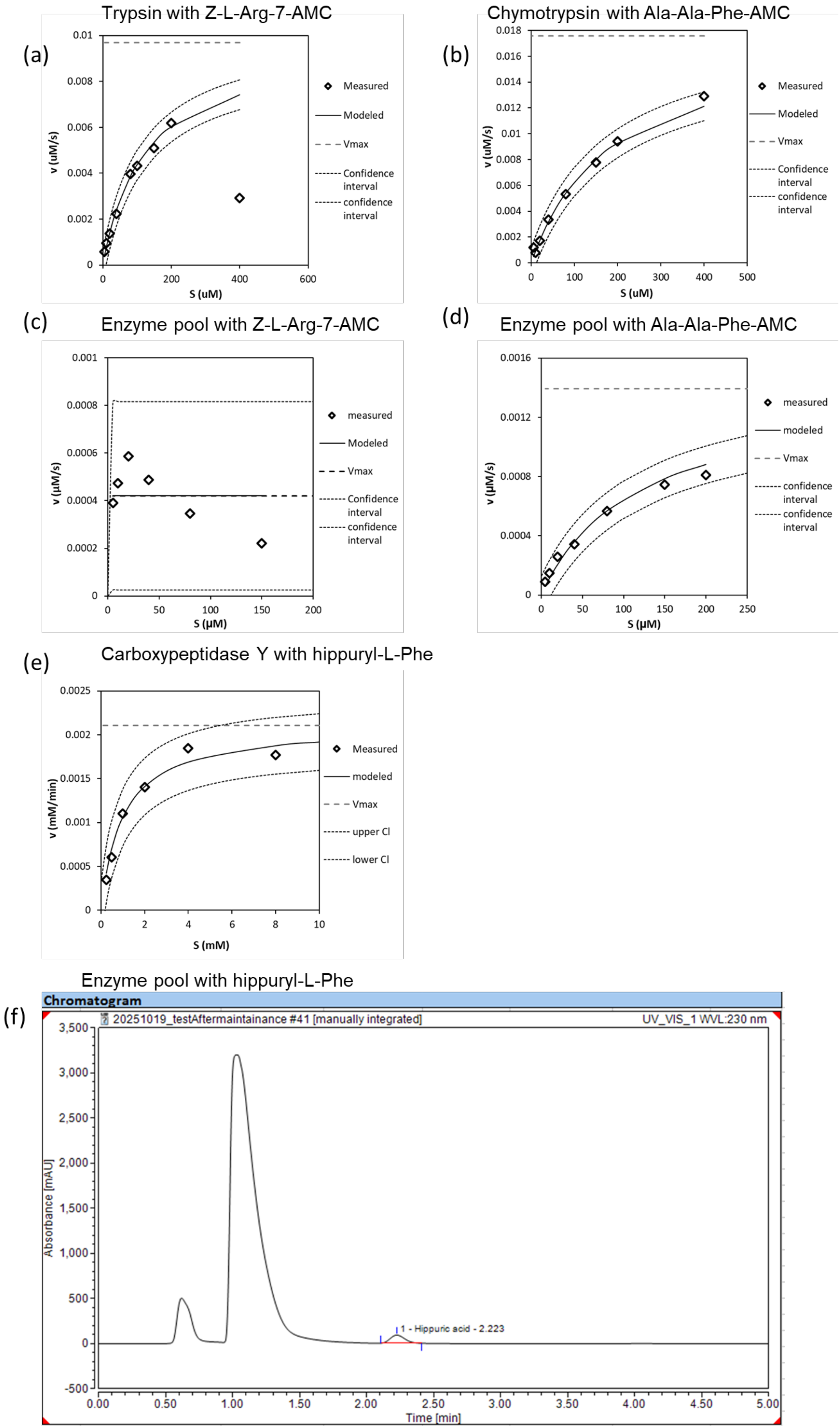

Integration Results

No.	Peak Name	Retention Time min	Area mAU*min	Height mAU	Relative Area %	Relative Height %	Amount
1	Hippuric acid	2.223	11.152	89.575	100.00	100.00	n.a.
Total:			**11.152**	**89.575**	**100.00**	**100.00**	

Figure S1. Michaelis-Menton kinetics of trypsin (50 nM, V_{max}= 0.0097 μM /min, K_m= 122.03 μM, (a)), chymotrypsin (0.005 U/mL, V_{max}= 0.0176 μM /min, K_m= 179.65 μM, (b)), and carboxypeptidase Y (0.5 U/mL, V_{max}= 0.0021 μM /min, K_m= 0.99 mM, (e)). Kinetics of the aquatic enzyme pool for the model substrate of trypsin (c), the curve did not follow typical Michaelis-Menten kinetics, the highest measured rate was 0.0005872 μM /s when the substrate was 20 μM; for the model substrate of chymotrypsin (d), yielding V_{max}= 0.001391 μM /s, K_m = 115.99 μM. The chromatogram of the product hippuric acid after incubating hippuryl-L-Phe with the aquatic enzyme pool for 4 hours (the reaction rate was too slow to draw Michaelis-Menton kinetics).

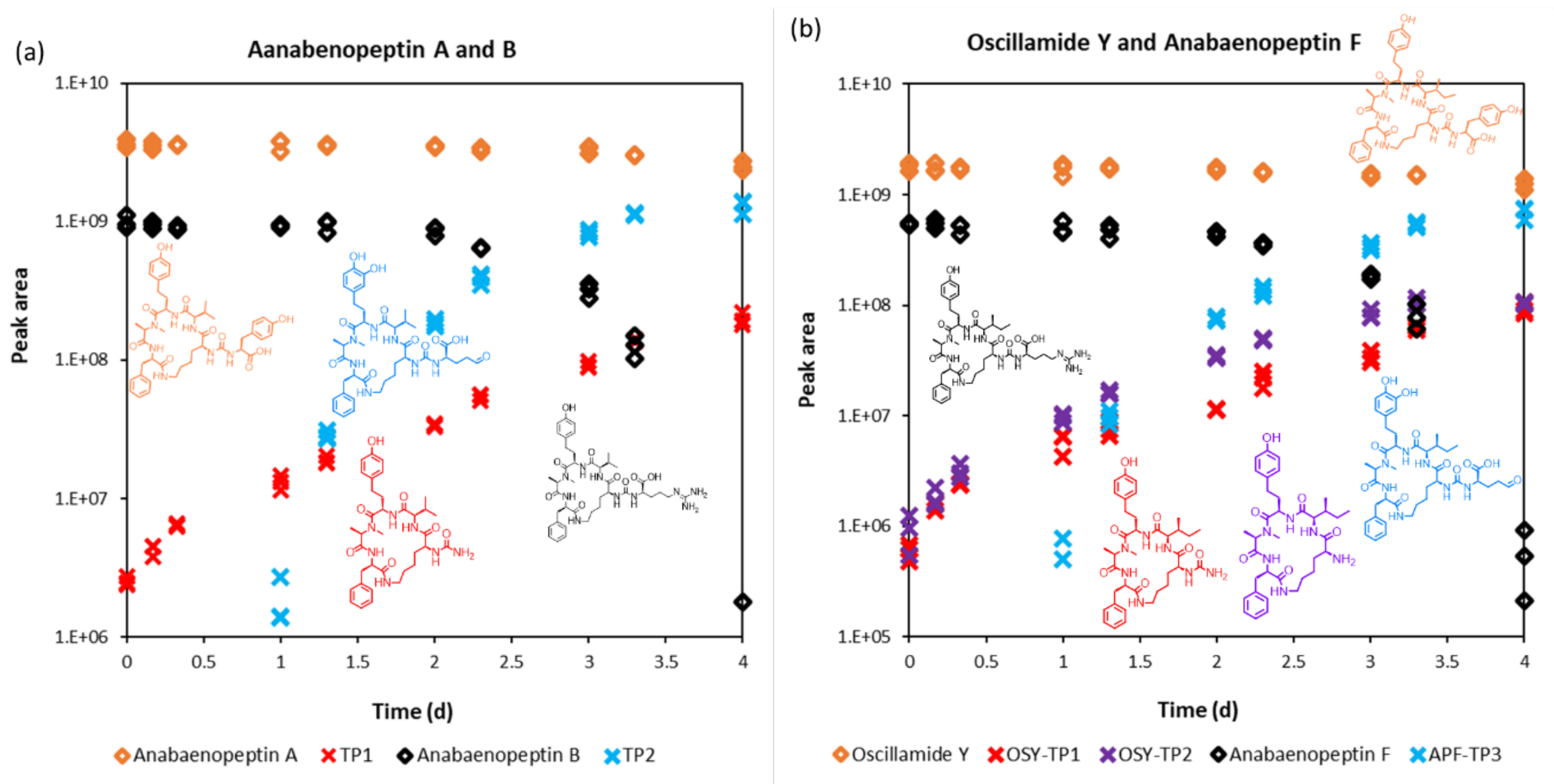

Figure S2. Decay kinetics of anabaenopeptin A and anabaenopeptin B (a), oscillamide Y and anabaenopeptin F (b), increase of their transformation products, and metabolite structures. Note that the biofilm enzyme pool was reacted with cyanobacterial extracts not with fractions, thus anabaenopeptin F presented here.

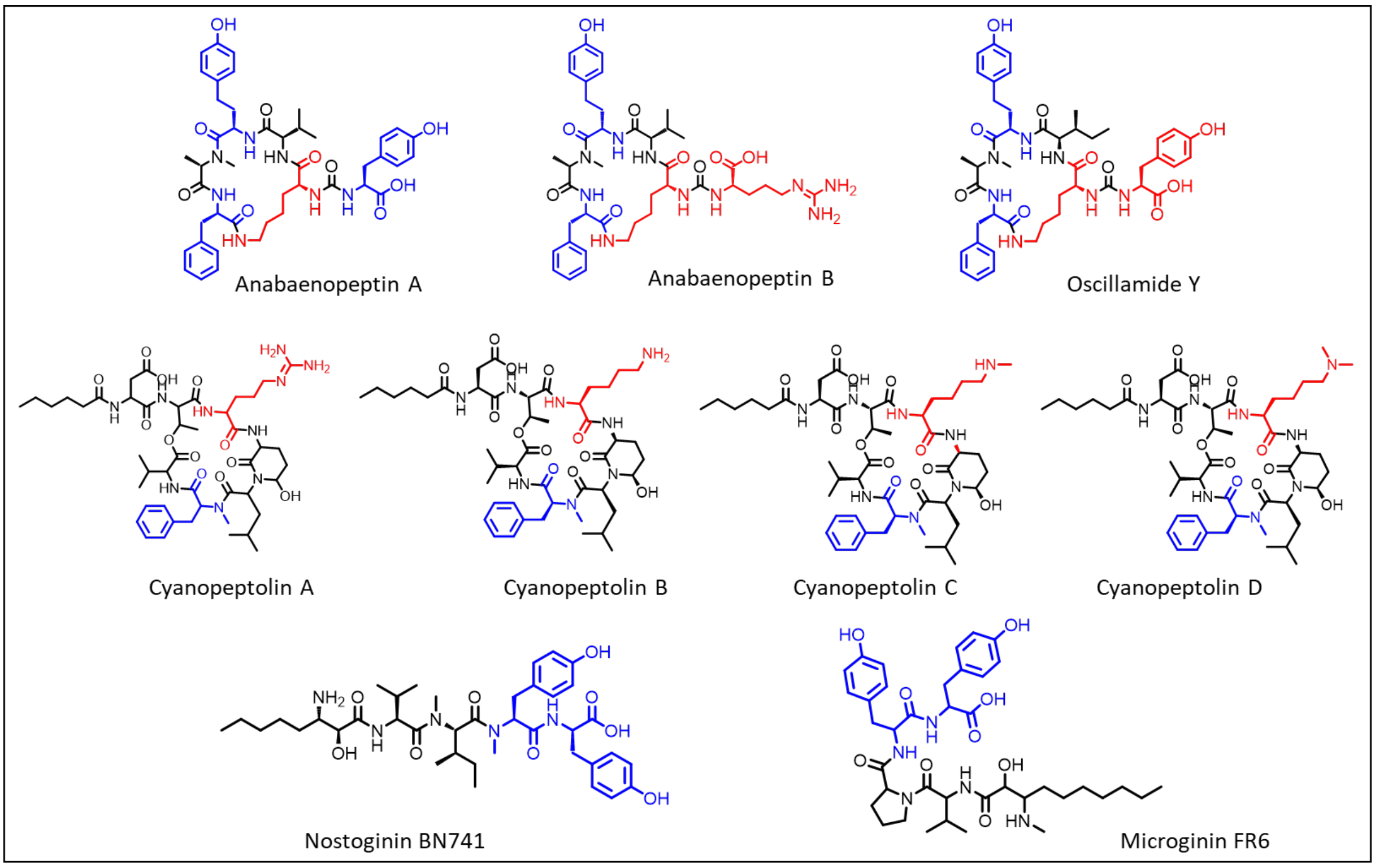

Figure S3. Structures of tested cyanopeptides, basic amino acid residues are highlighted in blues (arginine and lysine), aromatic amino acid residues are highlighted in red (phenylalanine and tyrosine)

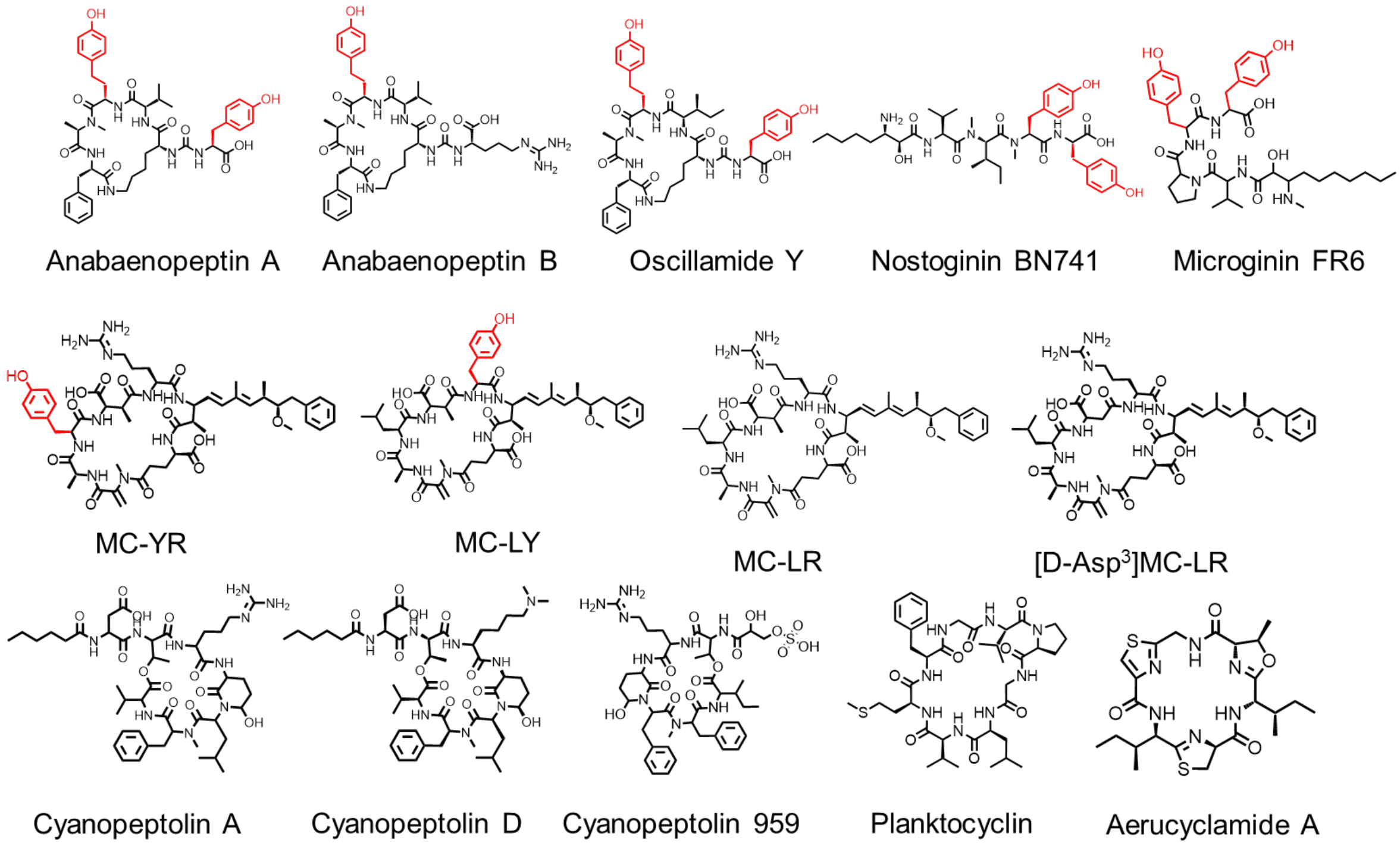

Figure S4. The structures of tested cyanopeptides for MCO1, the tyrosine (tyrosine-like) residues are highlighted.
